# AI‐Assisted Self‐Powered Wearable Dual‐Mode Sensor With TENG and Stretchable Optical Fiber for Neurological Disorder Diagnostics

**DOI:** 10.1002/advs.202522179

**Published:** 2026-03-12

**Authors:** Tianliang Li, Han Liu, Guoxu Liu, Qian'ao Wang, Haotian Zhou, Guiyi Liu, Yan Xu, Jun Wang, Zuqiang Wang, Feng Zhu, Feiling Luo, Zhong Lin Wang, Chi Zhang

**Affiliations:** ^1^ School of Mechanical and Electronic Engineering Wuhan University of Technology Wuhan Hubei China; ^2^ Beijing Key Laboratory of High‐Entropy Energy Materials and Devices Beijing Institute of Nanoenergy and Nanosystems Chinese Academy of Sciences Beijing P. R. China; ^3^ School of Nanoscience and Engineering University of Chinese Academy of Sciences Beijing P. R. China; ^4^ School of Information Engineering Wuhan University of Technology Wuhan Hubei China; ^5^ Department of Neurology Union Hospital Affiliated to Tongji Medical College Huazhong University of Science and Technology Wuhan Hubei China; ^6^ Renmin Hospital of Wuhan University Wuhan Hubei China; ^7^ Department of Encephalopathy II Yangxin County Traditional Chinese Medicine Hospital Huangshi Hubei China; ^8^ Department of Neurology Yangxin County People's Hospital Huangshi Hubei China

**Keywords:** AI‐assisted diagnosis, dual‐mode sensor, self‐powered, triboelectric nanogenerator

## Abstract

Wearable sensors hold significant potential for managing lower‐limb dysfunction in neurological disorders, but current systems remain constrained by unimodal sensing, external power dependence, and limited diagnostic capabilities. Here, we present a wireless wearable dual‐mode sensor (WDMS) integrating three polyurethane‐based flexible optical strain (PFOS) components with a contact‐separation mode triboelectric nanogenerator (CS‐TENG). The PFOS components are used for muscle signal monitoring, while the CS‐TENG simultaneously monitors plantar pressure and harvests biomechanical energy to power the WDMS, eliminating external power dependence. Furthermore, leveraging gait data from 60 individuals, an embedded Convolutional Neural Network–Long Short‐Term Memory (CNN‐LSTM) model achieved 94.23% accuracy in distinguishing Parkinson's disease (PD) and stroke, while quantitatively evaluating rehabilitation progress after pharmacological and physical therapy interventions. By synergizing multimodal sensing, AI‐driven analysis, and clinical validation, this technology advances beyond passive monitoring to provide intelligent diagnostic support. Its self‐sufficiency and scalability facilitate transformative home‐based management of neurological disorders.

## Graphical abstract

This manuscript presents the WDMS platform, an AI‐assisted, self‐powered wearable dual‐mode sensor for tele‐neurology. It integrates a contact–separation TENG insole with stretchable polyurethane optical‐fiber strain sensors to synchronously track plantar pressure and lower‐limb muscle deformation. Multimodal features drive CNN–LSTM recognition of Parkinson's and post‐stroke gait, enabling home monitoring and feedback‐guided rehabilitation.

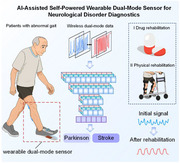

## 1 Introduction

Neurodegenerative disorders such as stroke and Parkinson's disease (PD) frequently impair the coordinated activity of the skeletal, muscular, and nervous systems [1, 2], characterizing shuffling steps, asymmetrical stride length, and diminished joint mobility [3]. Real‐time monitoring of lower‐limb joint kinematics and muscle dynamics is essential [4] for facilitating early diagnosis, furnishing valuable biomarkers for tracking disease progression, and evaluating rehabilitation efficacy [5]. Among monitorable metrics, strain sensing plays a critical role in capturing joint motion and muscle activation, offering direct, quantifiable insights into biomechanical performance [6, 7, 8]. Conventional gait analysis tools—including optical motion capture systems and instrumented walkways—suffer from high costs, bulkiness, and confinement to laboratory settings, rendering them unsuitable for continuous real‐world monitoring [9, 10]. Wearable sensors offer a promising alternative, facilitating long‐term ambulatory biomechanical surveillance [11, 12]. Commercial and research‐grade gait wearables based on inertial measurement units (IMUs), resistive/capacitive pressure insoles, and surface electromyography (sEMG) have enabled out‐of‐lab assessment of motor function, yet they remain constrained by rigid or bulky form factors, wired or frequently recharged power supplies, and susceptibility to motion artefacts and electromagnetic interference [13, 14, 15]. Driven by this need, substantial progress has been achieved in flexible and stretchable electronic materials for sensor design [16, 17]. Yet conventional electronic sensors relying on piezoelectric, capacitive, or resistive mechanisms [18, 19, 20, 21, 22, 23] face persistent challenges: device miniaturization constraints, current leakage from inadequate insulation, and vulnerability to electromagnetic interference (EMI) [24, 25]. Quartz optical fibers possess inherent advantages of electrical safety, EMI immunity, and compactness [26, 27, 28], which still exhibit limited stretchability (strain limit < 1%) [29] and are difficult to accommodate large joint deformations. Polyurethane (PU) optical fiber features flexibility, mechanical robustness, and excellent optical properties [30, 31], emerging as an ideal candidate for wearable sensors with human body strain sensing. However, most wearable sensors still rely on wired transmission with external powered demodulation units, which restrict daily mobility. Moreover, prevailing unimodal sensing captures only fragmented dimensions of gait dynamics, which is insufficient for decoding complex movement patterns [32].

Triboelectric nanogenerators (TENGs), which harness contact electrification and electrostatic induction, have emerged as a promising technology for converting ambient mechanical energy and human motion into electrical signals [33, 34, 35, 36]. Therefore, TENG‐based self‐powered sensors hold great promise for health monitoring and rehabilitation [37, 38]. Recent triboelectric smart socks, shoes, and insoles have realized self‐powered plantar pressure sensing, gait analysis, and human–machine interfaces in healthy participants and mimicked pathological gait, highlighting the potential of TENG platforms for ambulatory gait monitoring and wearable health applications [39, 40, 41, 42]. However, these systems typically focus on single‐site plantar signals, depend on externally powered electronics for multi‐channel acquisition and wireless transmission, and rarely capture multi‐site muscle dynamics or directly target neurological cohorts. Integrating TENG technology allows for the development of a stable dual‐mode sensing, which not only enhances robustness against environmental interference [43] but also enriches data features for advanced analytics [44]. However, the lack of embedded diagnostic intelligence [45] makes the wearables remain merely a passive data recorder, difficult to improve the level of intelligence further. The recent rapid evolution of artificial intelligence (AI), particularly deep learning, is now transforming wearables into active diagnostic platforms capable of real‐time disease detection and rehabilitation assessment [46]. Machine learning models can fully leverage multimodal sensor data to achieve tasks such as disease detection [47], progression monitoring, and rehabilitation outcome prediction [48]. Embedding AI‐driven analytics into sensing systems elevates diagnostic precision while optimizing clinical workflows [49]. Thus, developing wearable systems that synergize superior wearability, dual‐modal sensing capabilities, and AI‐assisted diagnostics has become imperative for advancing precision healthcare and intelligent rehabilitation.

To address these challenges, this study presents a wearable dual‐mode sensor (WDMS) that integrates flexible strain sensing, self‐powered energy harvesting, and wireless gait recognition. Unlike previously reported dual‐mode or TENG‐based gait systems that usually monitor single‐site signals and rely on externally powered electronics, the WDMS is specifically architected around neurological gait biomechanics by combining a contact‐separation mode triboelectric nanogenerator (CS‐TENG) that simultaneously harvests energy and senses foot–ground loading with three PFOS components on key lower‐limb muscles and an AI‐enabled analysis pipeline that delivers clinically interpretable gait biomarkers and interactive human–machine interfaces. The PFOS components feature a tri‐layer structure consisting of a PU fiber sensing core seamlessly interfaced with plastic optical terminals. The PU fiber exhibits extraordinary elongation (> 900% strain), while a quantitative model based on Beer–Lambert's law correlates optical attenuation with tensile strain. Fabricated via a streamlined process, the PFOS components demonstrate excellent flexibility, high sensitivity (12.25 mV/%), mechanical resilience (up to 100% tensile strain), temperature adaptability (0–55°C), durability (>  20,000 cycles), and waterproofness. A switchable‐mode self‐powered circuit board enables dual functionality—sensing and energy storage—when coupled with the CS‐TENG. The proposed CNN–LSTM algorithm, based on gait data acquired from the WDMS, achieved an identification accuracy of 94.23% across a cohort of 60 subjects, including healthy individuals, stroke patients, and PD patients. This framework further quantifies rehabilitation outcomes under pharmacological treatments and assistive rehabilitation interventions, providing valuable insights for early neurological screening and rehabilitation assessment. Notably, the PFOS components translate joint kinematics into real‐time human‐machine interaction (HMI) commands, enabling hands‐free video control and wireless navigation of miniature vehicles‐enhancing patient engagement during rehabilitation. Collectively, the WDMS provides a scalable platform for remote health monitoring, intelligent diagnosis, and personalized rehabilitation—thereby profoundly improving outcomes and quality of life for individuals with neurological disorders.

## 2 Results

### 2.1 Function and Design of the WDMS

**FIGURE 1:**
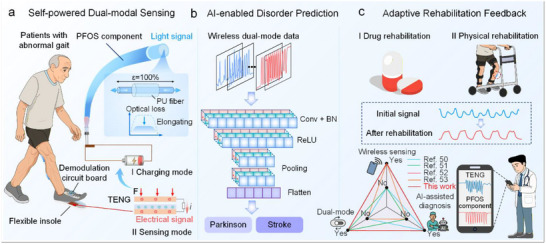
Function and design of the WDMS. (a) Working principles of PU fibers and integration with footwear to form a self‐powered WDMS using a CS‐TENG. (b) AI‐assisted remote gait recognition for disease diagnosis, involving wireless data transmission and neural network processing. (c) Monitoring and evaluation of rehabilitation scenarios, including comparison with prior related studies.

This work introduces a WDMS composed of three PFOS components and a CS‐TENG, as illustrated in Figure 1a. The CS‐TENG is embedded within a flexible insole and interfaced with a miniaturized demodulation circuit. During user locomotion, biomechanical energy from footsteps is harnessed and converted into electrical energy, charging a lithium battery and powering the system. Once sufficient energy is accumulated, a customized mobile application enables dynamic switching between the powering and sensing modes. In sensing mode, the CS‐TENG operates as a passive signal acquisition unit that captures gait dynamics, while the PFOS components quantify joint‐induced strain and wirelessly transmit the data to a host computer for further processing. By integrating these two sensing modules, we have developed a comprehensive remote healthcare monitoring platform. This system acquires multi‐site signals and wirelessly streams them to external devices or mobile applications for real‐time visualization and clinical evaluation. The bimodal data streams are processed through AI algorithms, which successfully identify pathological gait patterns in Parkinson's and stroke patients (Figure 1b). During rehabilitation, a custom mobile application quantitatively tracks gait metrics before and after pharmacological interventions and device‐assisted therapy, enabling physicians to objectively evaluate recovery progress (Figure 1c). Compared to prior efforts [50, 51, 52, 53], this system represents a significant advancement through the integration of wireless operation, dual‐modal sensing, and AI‐assisted diagnosis. Overall, this WDMS and its healthcare platform represent a transformative advancement in medical Internet of Things (IoT). By unifying materials, devices, hardware, and analytics into a cohesive framework, this system paves the way for scalable solutions in human‐environment interaction applications.

### 2.2 Construction of the WDMS

**FIGURE 2:**
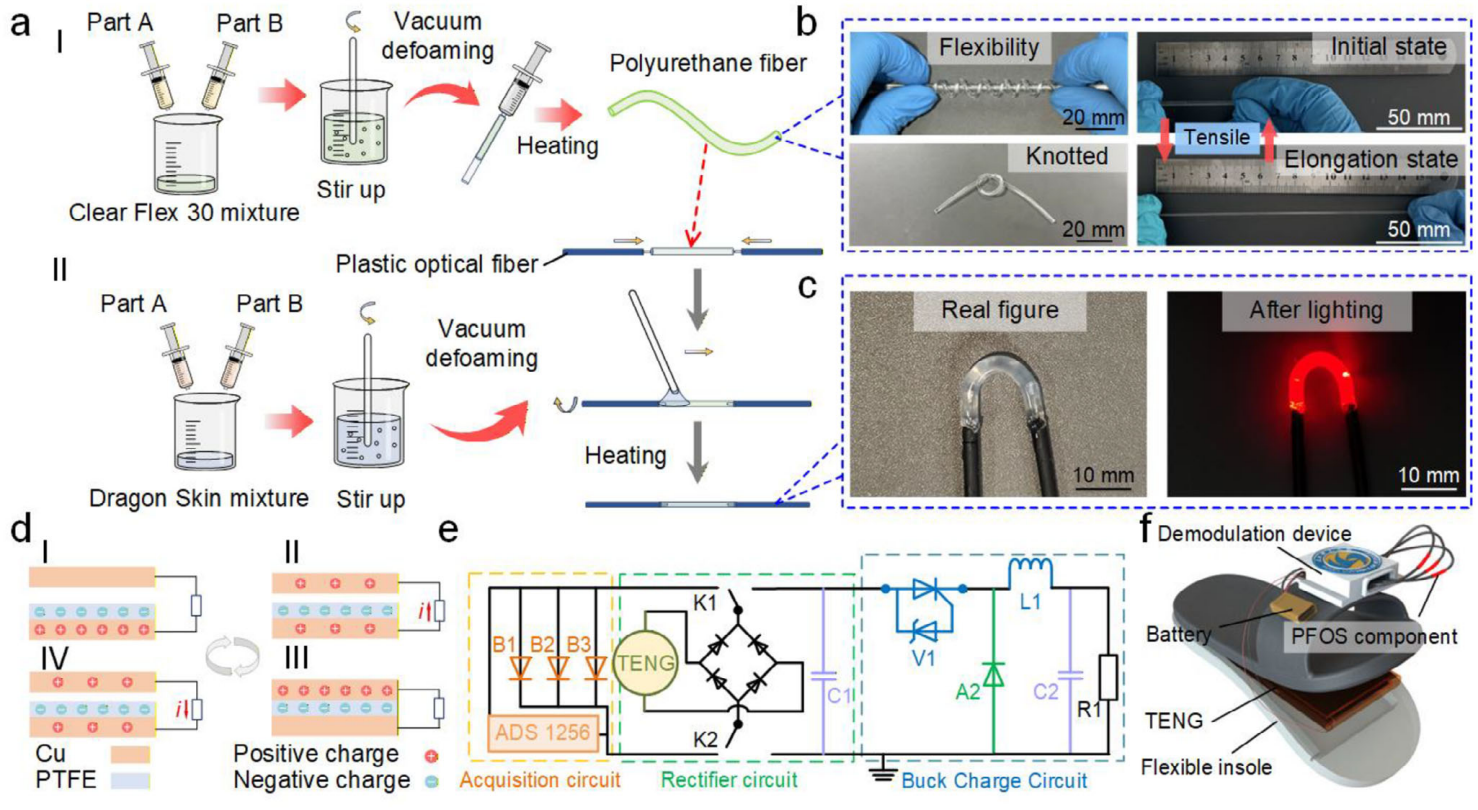
Construction of the WDMS. (a) Fabrication process of the PFOS component. (b) The flexibility and stretchability of the PU fiber. (c) Photograph of the PFOS component and its illuminated state. (d) Sensing schematic of the CS‐TENG. (e) Functional schematic of the switchable‐mode circuit board. (f) Schematic of the gait acquisition shoe.

The fabrication of the PFOS component encompasses two main steps: the preparation of the PU fiber and the assembly of the core‐clad structure. To synthesize the PU fiber, components A and B of Clear Flex 30 were mixed at a 1:1 ratio to form a homogeneous PU precursor. The mixture underwent vigorous mechanical stirring followed by vacuum degassing to eliminate entrapped air bubbles. The degassed precursor was then injected into pre‐cut polytetrafluoroethylene (PTFE) tubes via a syringe. After thermal curing (60°C, 5 h), the PTFE tube was mechanically peeled away, yielding a transparent, flexible PU fiber (Figure 2a). The resulting fiber exhibited remarkable flexibility, allowing easy bending and twisting, and could be stretched up to 300% without structural failure (Figure 2b). A red laser was injected into one end of the PU fiber and emitted from the other end to prove the good light‐guiding capability of the PU fiber (Figure S1). A fatigue test was conducted on a PU fiber (diameter: 2.0 mm, length: 51 mm) at 100% strain. After 1,000 cycles of repeated stretching (with measurements recorded every 100 cycles), the fiber's length change was only 1.96%, confirming its outstanding mechanical durability (Figure S2). For the core‐clad assembly, two stripped‐end plastic optical fibers (POFs) were axially aligned and inserted into opposing ends of an uncured PU fiber segment. Precise core‐centering was achieved through custom PTFE alignment jigs. The assembly was subsequently heated until the PU cured, resulting in a core‐only PFOS component. The cladding was fabricated using a similar method to Dragon Skin 20. After mixing components A and B, the mixture was degassed and evenly coated onto the PU fiber using a glass rod. Once cured at room temperature, the final PFOS component with a core‐cladding structure was obtained (Figure 2c). Scanning electron microscopy (SEM) images confirmed the clear interface between the fiber core and the cladding (Figure S3). Biocompatibility considerations informed this layer assignment. A prior study performed ISO 10993–5 cytotoxicity testing (L‐929, MTT, 37°C/24 h extraction) on these elastomers and reported that Dragon Skin met the 70% viability criterion (e.g., 85.3% viability in the 100% extract), whereas Clear Flex 30 showed reduced viability below the criterion under high‐concentration extracts; moreover, MSDS information indicates that uncured Clear Flex 30 constituents may cause irritation/sensitization [54]. Accordingly, in our design, fully cured Clear Flex 30 is used as the core, while Dragon Skin 20 serves as the outer cladding/skin‐contacting encapsulation layer to physically isolate the core from direct skin exposure.

The CS‐TENG was fabricated through a simple three‐step process: copper foil electrodes were laminated onto acrylic substrates with embedded leads, a PTFE film was applied as the triboelectric layer, and the assembly was encapsulated with Kapton film to ensure structural integrity (Figure S4). Triboelectric charges are generated when the copper electrode on the upper layer contacts and separates from the PTFE membrane on the lower layer. Upon separation, electrostatic induction creates a potential difference between the electrodes, driving current through an external circuit to achieve energy harvesting (Figure 2d). A miniaturized circuit board was designed to support two operational modes: charging mode and sensing mode. To evaluate the CS‐TENG's charging capability, capacitors with different specifications were connected to assess their charging performance. The results showed that the 10 µF capacitor reached its maximum voltage under the same pressing amplitude and duration, but it remained below the 3.3 V required for system operation (Figure S5a). By integrating a power management circuit (PMC), the 10 µF capacitor achieved the target 3.3 V within 15 s (Figure S5b), with subsequent discharge characteristics quantified at varying voltages (Figure S6). In sensing mode, the CS‐TENG functions as a pressure‐sensitive sensing unit (Figure 2e). Mode switching can be achieved via a physical button or remotely through a mobile application. By embedding the CS‐TENG into a flexible insole made of PU material and fixing the demodulation circuit board and battery on the upper surface of the shoe, a gait acquisition shoe was prepared (Figure 2e and Figure S7). Because the CS‐TENG is fully encapsulated within the PU insole and electrically isolated from the wearer, no electrode is in direct contact with the skin, thereby ensuring safety during prolonged wear.

### 2.3 Performance Characterization of the WDMS

When integrated with the circuit board, the PFOS component guides light emitted by the on‐board source through a POF into the PU fiber, and the transmitted intensity is quantified by a photodiode. Under tensile strain, the detected intensity decreases due to (i) strain‐induced scattering and microstructural perturbations within the PU waveguide and (ii) imperfect mode coupling at the POF–PU interfaces. In a general form, the strain‐dependent transmission can be written as:

(1) $$\begin{array}{cccc} & I\;\left(\varepsilon \right)=I_{0}\;\;\eta_{1}\left(\varepsilon \right)\;\eta_{2}\left(\varepsilon \right)\;\exp\left(-\int_{0}^{L\left(\varepsilon \right)}\alpha \left(z,\varepsilon \right)\;dz\right) & & \end{array}$$

where *I*_0_is the incident optical power, η_1_(ε)and η_2_(ε)denote the strain‐dependent input/output coupling efficiencies, *L*(ε)is the strained length of the PU fiber, and α(*z*, ε)is the distributed attenuation coefficient including both intrinsic loss and mechanically induced loss.

For practical calibration and real‐time demodulation, we adopt an effective Beer–Lambert‐type relationship by assuming (i) approximately uniform strain over the PU section so that α(*z*, ε) ≈ α_0_ + *k_s_* ε, and (ii) the POF ends are pre‐aligned and mechanically stabilized, making η_1_and η_2_only weakly dependent on strain within the operational range. In addition, for the lumped model, we treat *L*(ε)as a nominal effective length *L*, which yields

(2) $$\begin{array}{cccc} & I\left(\varepsilon \right)=I_{0}\;\;\eta_{1}\;\eta_{2}\;\exp\left[-\left(\alpha_{0}+k_{s}\varepsilon \right)L\right] & & \end{array}$$

where α_0_is the intrinsic loss coefficient and *k_s_* is the effective extinction coefficient capturing strain‐induced attenuation. Taking the natural logarithm of Equation (2) gives a first‐order linearized form:

(3) $$\begin{array}{cccc} & \ln\;I\left(\varepsilon \right)=C-k_{s}L\varepsilon & & \end{array}$$

where $C=\ln\left(I_{0}\eta_{1}\eta_{2}\right)-\alpha_{0}L$. Therefore, the optical attenuation increases monotonically with tensile strain, and ln *I*decreases approximately linearly with εin the calibrated regime.

At higher strains, deviations from Equation (2) may emerge because η_1_(ε)and η_2_(ε)can become strain‐dependent (interfacial mismatch/alignment changes), and α(*z*, ε)can exhibit nonlinear growth due to Poisson contraction–induced modal redistribution, enhanced scattering from microstructural evolution, and viscoelastic relaxation. In such cases, a second‐order extension (e.g., $\ln I=C-a_{1}\varepsilon -a_{2}\varepsilon^{2}$) can be used if needed; in this work, we restrict the operational strain window to maintain near‐linear transduction.

To determine the optimal fiber diameter, PU fibers with diameters of 2.0, 2.5, and 3.0 mm were fabricated and mechanically characterized using a Sunrise Instruments (SRI) multi‐axis force sensor. Results showed that the 2.0 mm fiber achieved ultrahigh stretchability, sustaining strain exceeding 900% without structural failure (Figure S8). Consequently, this diameter was selected for the PFOS component. To ensure robust interfacial mechanics at the plastic–PU junction, the operational strain range was limited to 0–100%, which fully covers the strain levels expected in wearable applications. This range provides a conservative margin over physiologically relevant deformations encountered during daily gait [55, 56].

**FIGURE 3:**
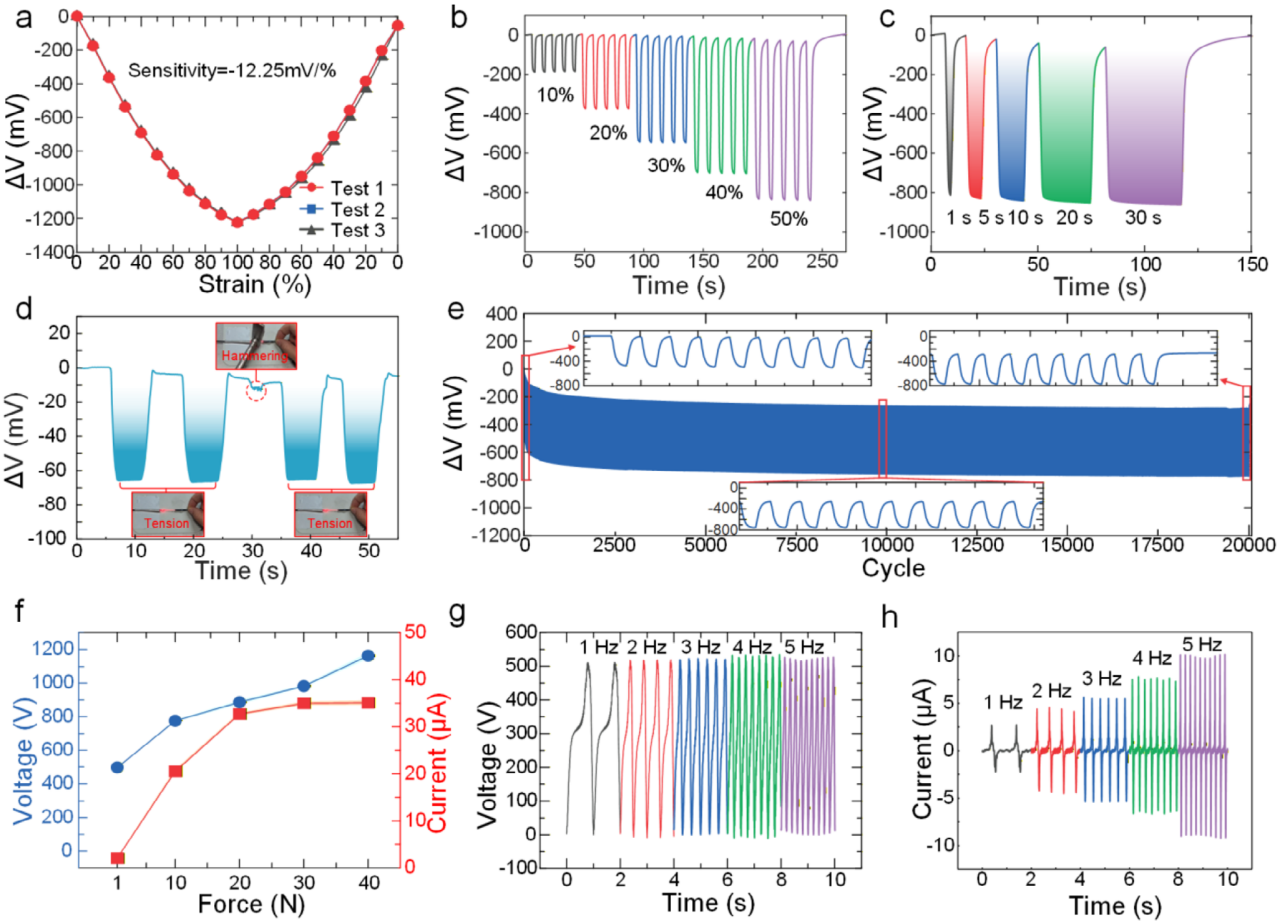
Performance characterization of the PFOS component and CS‐TENG. (a) Static calibration curve of the PFOS component. (b) Response of the PFOS component to the varying strain amplitude test. (c) Response of the PFOS component to varying durations at 50% strain. (d) Impact response test of the PFOS component. (e) Fatigue stability of the PFOS component under cyclic uniaxial tensile loading between 0% and 20% strain, recorded 20,000 cycles; the subplot illustrates the specific changes in sensor signals over different time periods. (f) Voltage and current output of CS‐TENG under different contact forces. (g) Voltage output of the CS‐TENG at different operating frequencies. (h) The current output of the CS‐TENG at different operating frequencies.

The PFOS component was calibrated on a computer‐controlled stepper motor platform, undergoing cyclic tensile loading from 0% to 100% strain at 10% increments across three repeated tests (Figure 3a and Figure S9). The measured sensitivity was 12.25 mV/%, with a linearity of R^2^ = 0.96 and a hysteresis error of 4.62%. To ensure quantitative traceability of hysteresis, we explicitly define the maximum hysteresis error as:

(4) $$H\;\left(\%\right)=\frac{max_{\varepsilon }|V_{\mathrm{load}}\left(\varepsilon \right)-V_{\mathrm{unload}}\left(\varepsilon \right)|}{V_{\mathrm{FS}}}\;\times 100\%$$

where *V*_FS_ =  ∣*V*_load_(100%) − *V*_load_(0%)∣denotes the full‐scale output span. Based on the stepwise mean values averaged over three tests. The maximum hysteresis gap occurs at ε  =  10% with Δ *V*_max_ =  56.69 mV, corresponding to *H*_max_ =  4.63%; this strain point has been explicitly marked in the calibration figure (Figure S10). Dynamic tests included five cycles of repeated loading from 10% to 50% strain (Figure 3b), holding tests at 50% strain for various durations (1 to 30 s) (Figure 3c), and different intervals (8.5, 5.5, and 1.5 s) (Figure S11) to assess signal stability. The PFOS component also demonstrated reliable performance under increasing and decreasing strain cycles (Figure S12) and showed a fast response time (Figure S13). Furthermore, a series of experiments was conducted to systematically evaluate the environmental adaptability of the PFOS component. Specifically, after subjecting the PU fiber segments of the PFOS component to impact testing, their output signals remained normal (Figure 3d). Moreover, when the signal‐transmitting optical fibers were bent to different radii (30 mm, 6 mm) (Figure S14), immersed in water (Figure S15), or subjected to temperature variations from 0°C to 55°C (Figure S16), the output signals of the PFOS components exhibited only minor fluctuations. To further assess real‐life environmental stability, we additionally performed repeated dry–wet cycling (alternating dry stretching and stretching under water immersion for five rounds), simulated‐sweat exposure using 0.4 wt.% saline with intermittent stretching up to 12 h, prolonged skin‐contact evaluation using a PU elastomeric film on dorsal hand skin for 0–8 h, and temperature cycling between 20 and 60°C for three cycles; in all cases, the PFOS signals remained stable with negligible drift and reversible temperature‐dependent variations (Figures S17–S20). These results further confirm the robustness and reliability of the component. The long‐term stability test is another important indicator for evaluating the PFOS component. Subjected to 20,000 fatigue cycles testing at 20% strain, the PFOS component exhibited no observable optical signal distortion (Figure 3e). Notably, the long‐cycle fatigue response can still exhibit a slowly evolving envelope/baseline, which is consistent with viscoelastic stress relaxation and creep in elastomeric polymers. Together with the loading–unloading mismatch in calibration, we attribute the hysteresis primarily to intrinsic PU viscoelasticity (path‐dependent response due to chain rearrangement and internal dissipation) and interfacial effects at the plastic‐fiber–PU/PFOS junction (shear deformation within the bonding layer or micro‐slippage that yields different coupling gains along the loading versus unloading paths). Moreover, the hysteresis is more pronounced at low strain (10–20%), consistent with higher sensitivity to interfacial seating/friction and viscoelastic relaxation during the initial loading regime. This exceptional endurance—surpassing state‐of‐the‐art stretchable sensors [57, 58, 59, 60, 61, 62, 63] in both mechanical resilience and cyclic stability (Figure S21)—establishes a new benchmark for wearable photonic strain sensors. In addition, to evaluate potential photo‐oxidative aging/yellowing of the PU core under illumination, we continuously exposed the PFOS component to the LED for 21 days and monitored the 0% strain baseline; the maximum baseline fluctuation was 44 mV from an initial 2758 mV, corresponding to a maximum error of only 1.6% (Figure S22).

The output performance of the CS‐TENG was systematically characterized under variable contact forces and operating frequencies. Under a fixed frequency of 1 Hz, both short‐circuit current and open‐circuit voltage escalated monotonically with increasing contact force—from 2.1 µA and 500 V at 1 N to 35 µA and 1150 V at 40 N, respectively (Figure 3f). Under a constant contact force of 1 N, elevating the frequency from 1 to 5 Hz induced a 3.7‐fold surge in short‐circuit current, while the open‐circuit voltage remained stable (Figure 3g,h). The maximum output power reached 0.25 W across a 1 MΩ resistive load (Figure S23). Stability and durability tests showed that the CS‐TENG output remained stable under variable frequencies at constant force. Under different humidity conditions, the open‐circuit voltage decreased from 410 V (30% RH) to 330 V (75% RH), and the short‐circuit current dropped from 10.5 to 7.2 µA (Figure S24). Long‐term operation of the CS‐TENG at 3 Hz for more than 1 h revealed stable signal output and excellent device reliability (Figure S25).

### 2.4 WDMS for Measuring Multiple Parts of the Human Body

**FIGURE 4:**
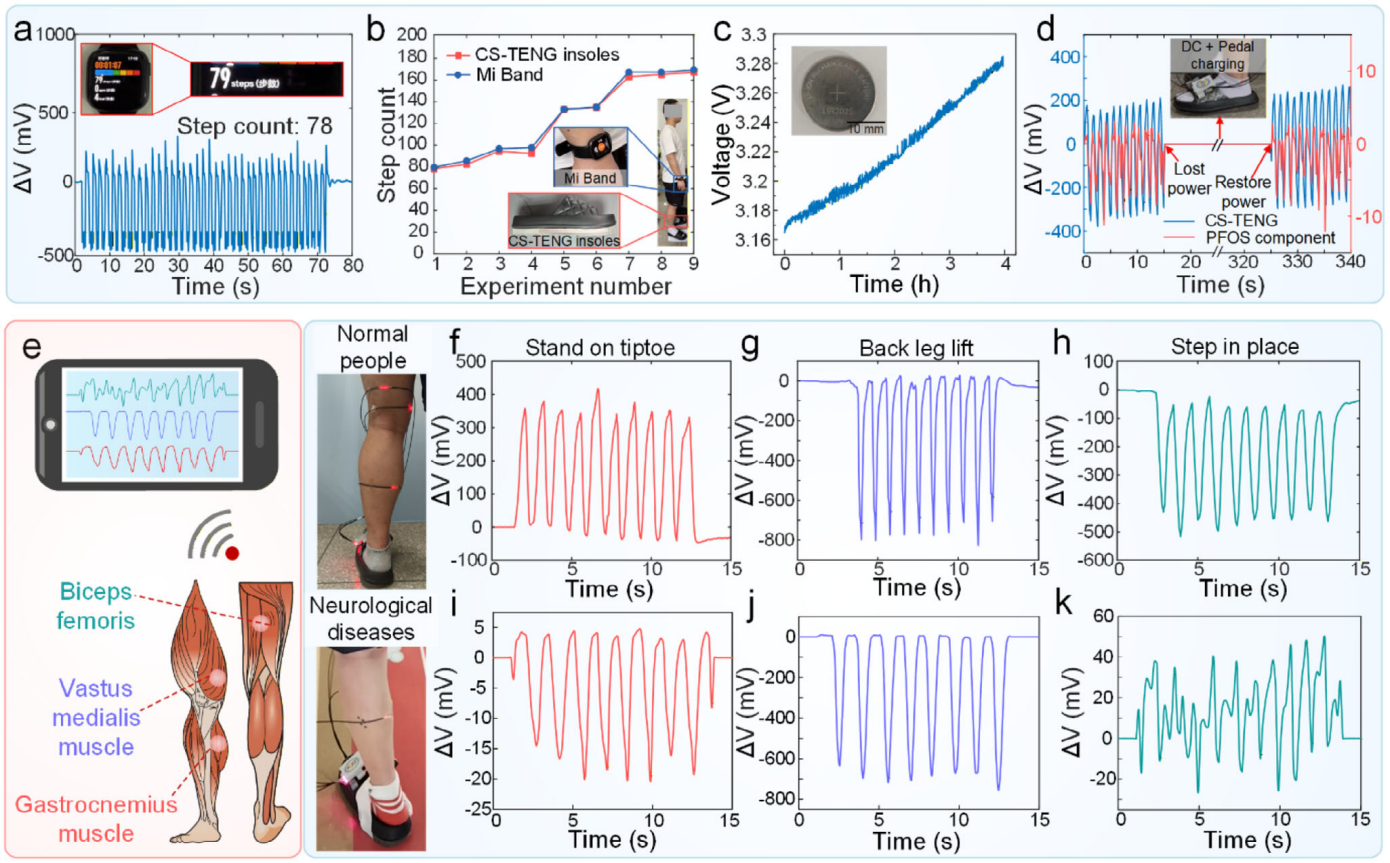
Application of the WDMS for multi‐site human body monitoring. (a) Comparison of gait signals recorded by the CS‐TENG component and a commercial wristband. (b) Results of nine comparative tests, including an experimental schematic. (c) Charging performance of a 25 mAh battery under the switchable‐mode circuit in energy storage configuration, the illustration is a physical picture of the battery. (d) Schematic of the system recovering power after DC and pedal charging when the WDMS is out of power. (e) Schematic of PFOS components integration with the APP operation for leg muscle monitoring. (f‐k) Strain signal detection at various lower limb muscle sites: (f) Gastrocnemius (healthy subject), (g) Vastus medialis (rear leg elevation, healthy subject), (h) Biceps femoris (walking, healthy subject), (i) Gastrocnemius (patient gait), (j) Vastus medialis (patient gait), (k) Biceps femoris (patient gait).

To verify the gait acquisition capability of the integrated gait acquisition shoe, we first evaluated the signal fidelity of the CS‐TENG embedded within a flexible insole. A volunteer wearing a commercial Xiaomi fitness tracker performs a stepping task while the step counts recorded by the tracker were cross‐validated against CS‐TENG signals (Figure 4a). Accounting for inter‐individual gait variability, eight additional trials encompassing diverse stepping regimes—including slow and fast walking—were conducted (Figure 4b and Figure S26). The CS‐TENG demonstrated exceptional step‐counting accuracy, with a maximum relative error of merely 5.49% compared to the commercial device (Figure S27).

The CS‐TENG dual‐mode operational flexibility was further verified. When switching from sensing to charging mode, biomechanical energy harvested during stepping replenishes the system. In charging mode, pressing the CS‐TENG continuously charges the onboard lithium battery (25 mAh). After 4 h of charging, the battery voltage increases from 3.165 to 3.28 V, storing 95.7 mWh of energy (Figure 4c). A custom mobile application enables real‐time Bluetooth transmission and seamless switching between sensing/charging modes. When system energy depletes, the WDMS can be reactivated via stepping‐induced energy harvesting or direct current charging, resuming normal operation within minutes (Figure 4d and Video S1). To clarify the real‐world energy balance, we characterized the full system power budget under an “acquire–upload–sleep” intermittent strategy (Figure S28 and Table S2). Specifically, at a rated system voltage of 3.2 V, the operating current is ∼0.2 mA during local data acquisition, ∼1.8 mA during Bluetooth data upload, and ∼0.007 mA in sleep mode (Figure S26). For a representative 10‐min operating cycle (480 s acquisition + 20 s upload + 100 s sleep), the total energy consumption is 424.64 mJ (Table S2). On the generation side, impedance matching indicates an effective output power of 3.92 mW for the CS‐TENG (Figure S29). Assuming a single‐step contact duration of ∼0.25 s and an overall rectification/regulation efficiency of 72%, the harvested energy is ∼0.7056 mJ per step, corresponding to ∼705.6 mJ over 1,000 steps (10 min of typical walking), which exceeds the above consumption with a net surplus of ∼280.96 mJ. Consistent with this system‐level budget, the battery voltage shows a slight increase after a 10‐min intermittent operating period (Figure S30). Therefore, under typical daily use that includes regular walking bouts and duty‐cycled sensing/Bluetooth transmission, the harvested energy can sustain the system, whereas prolonged sedentary periods or continuous high‐duty streaming may still require occasional supplemental charging; the app‐controlled mode switching is designed to adapt the duty cycle accordingly.

The PFOS components conform seamlessly to complex anatomical contours, enabling high‐fidelity detection of both subtle and large‐scale strains induced by muscle movements across diverse leg regions (Figure 4e). Acquired signals are wirelessly streamed in real‐time via a customized mobile phone app (Video S2). For lower limb assessment, PFOS components were first deployed on three critical calf muscle groups in a healthy volunteer. Signal responses were recorded from the gastrocnemius muscles during 10 heel raises (Figure 4f), the vastus medialis muscle during 10 leg raises (Figure 4g), and the biceps femoris muscle during 10 stationary steps (Figure 4h). The experiments found that the PFOS components accurately recorded motion states across distinct muscle groups and activities, demonstrating exceptional multi‐motion adaptability. Additional validation during walking and toe raises revealed stable signal kinetics in the vastus lateralis and tibialis anterior muscles (Figures S31 and S32). Finally, patients with neurodegenerative disorders were asked to wear slippers, and signal data were collected from the gastrocnemius muscle (Figure 4i), vastus medialis muscle (Figure 4j), and biceps femoris muscle (Figure 4k) during ambulation. Comparative analysis with healthy baselines enables discrimination of disease‐specific gait patterns. In addition to the legs, some patients with neurodegenerative diseases require rehabilitation of their hands or other parts of their body. The highly sensitive PFOS components can also be used to capture the micro‐scale strains in hand joints or facial muscles. When the PFOS components are fixed onto the finger joints (Figure S33b), consistent signals are recorded across five bending cycles at variable angles. Similar results were observed in the wrist joint tests (Figure S33c), confirming the sensor's high sensitivity to hand articulation. Furthermore, the components were tested on the elbow joint, where they reliably captured signals during 10 consecutive cycles of extension and 90° flexion (Figure S33d), demonstrating their capability for upper‐limb motion tracking. To evaluate their performance in micro‐motion detection, the PFOS components were attached to the masseter muscle (face), throat, and flexor digitorum superficialis (hand). They accurately recorded muscle‐induced motions such as mouth opening/closing, swallowing, and hand clenching/relaxation (Figure S34). Additionally, when mounted onto an elastic waistband, the sensor successfully captured abdominal expansion and contraction during various breathing patterns (Figure S35). Beyond gait monitoring, these on‐body tests are proof‐of‐concept demonstrations highlighting conformability and multi‐scale strain‐sensing generality. In summary, the PFOS components deliver rapid, sensitive, and stable joint motion detection across anatomical regions, while the CS‐TENG provides clinically validated gait signal acquisition. Together, they establish a closed‐loop biomechanical monitoring framework for next‐generation wearable health platforms.

### 2.5 AI‐Assisted Identification of Neurological Diseases

**FIGURE 5:**
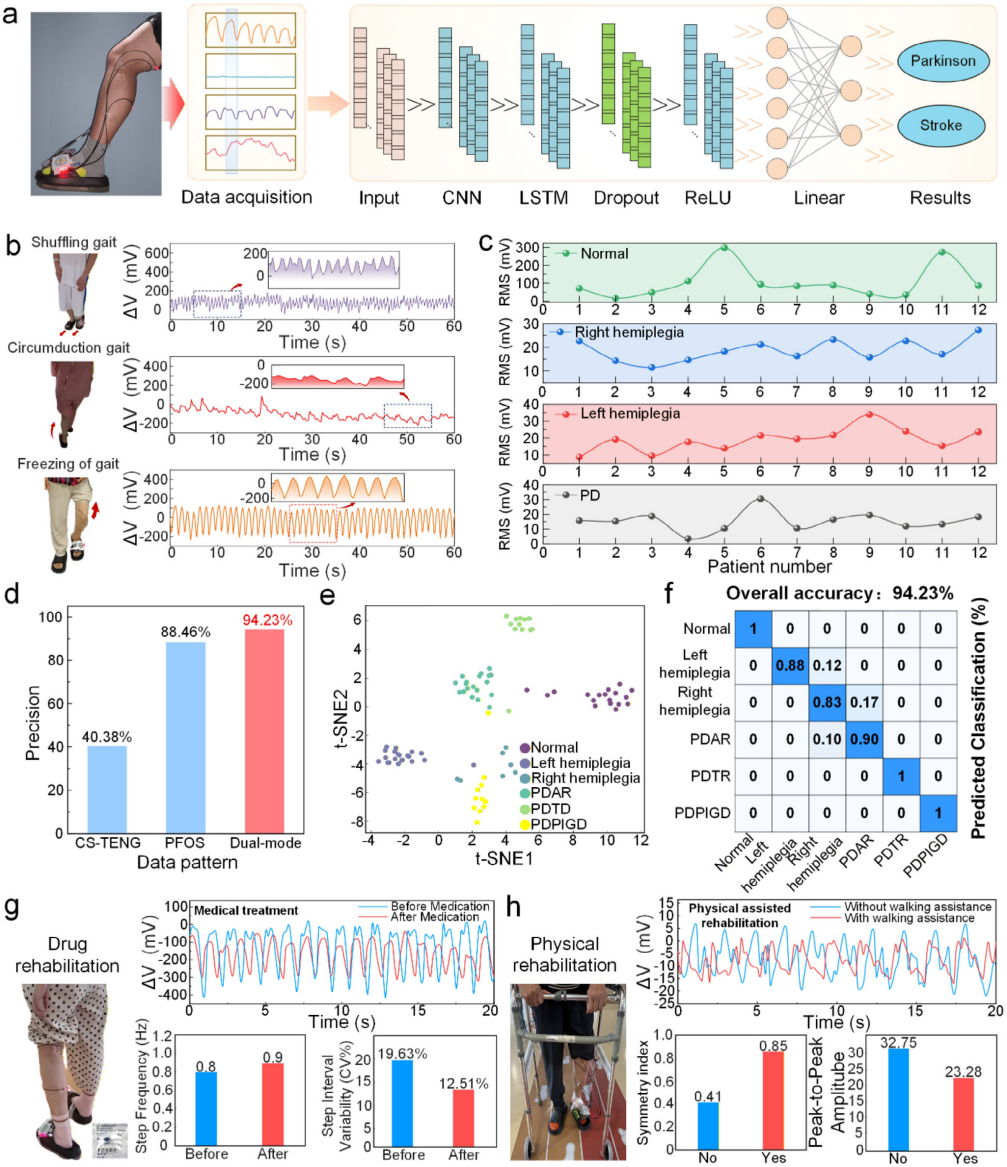
Neural network‐assisted gait analysis for neurological disorders identification. (a) Workflow of CNN‐LSTM‐based gait signal recognition for PD and stroke classification. (b) Waveforms illustrating gait signals associated with key gait symptoms. (c) RMS comparison across four subject cohorts. (d) Comparison of the accuracy of three sensing modalities. (e) t‐SNE visualization of feature distributions in six subject groups. (f) Confusion matrix for dual‐mode signals classification. (g) Pre‐ vs. post‐treatment gait signal comparison highlighting improvements in step frequency, amplitude, and stability. (h) Waveform analysis of unassisted versus assisted walking demonstrates enhanced gait symmetry and signal amplitude during rehabilitation.

Abnormal gait patterns characteristically manifest in patients with PD and post‐stroke hemiplegia. To achieve high‐precision classification of these neurological disorders, we integrated the WDMS with a Convolutional Neural Network‐Long Short‐Term Memory (CNN‐LSTM) neural network (Figure 5a). The system simultaneously acquires plantar pressure profiles and multi‐muscle strain signals from the lower limbs, enabling multi‐dimensional feature extraction through the CNN‐LSTM network to discriminate between PD and post‐stroke hemiplegia. Figure 5b presents representative pathological gait waveforms, including shuffling gait, circular stepping, and freezing of gait. Prior to clinical data collection, we conducted preliminary validation with healthy subjects performing four gait modalities: in‐place stepping, normal walking, stair ascent, and stair descent—confirming the system's robust responsiveness to kinematic transitions (Figure S36). Subsequently, an integrated gait shoe captured multi‐parametric biomechanical data from 60 individuals: 15 healthy controls, 30 stroke survivors (15 left/15 right hemiplegia), and 15 PD patients (Video S3). Root mean square (RMS) analysis of the gait signal revealed distinct intergroup signatures (Figure 5c): 12 healthy controls, 12 individuals with left hemiplegia, 12 with right hemiplegia, and 12 with PD. The results indicate significant intergroup differences in gait features, with individuals with PD exhibiting substantially lower RMS values, indicative of impaired motor function.

The multimodal gait dataset comprised four synchronized channels, including one plantar‐pressure signal (CS‐TENG) and three lower‐limb muscle‐strain signals (PFOS). Prior to being fed into the CNN‐LSTM, the PFOS channels were baseline‐corrected to suppress drift and low‐frequency motion artifacts by removing the DC offset (mean subtraction) and applying a fourth‐order zero‐phase Butterworth high‐pass filter. Subsequently, all four channels were robustly normalized using median and interquartile range scaling to mitigate outlier influence and harmonize amplitude ranges across participants. Continuous recordings were segmented into fixed‐length windows of 300 steps to form model inputs.

Given the moderate cohort size (n = 60), we explicitly evaluated generalizability using subject‐wise grouped cross‐validation at the participant level. In each fold, all windows from a given subject were assigned exclusively to either the training split or the held‐out test split (≈ 80%/20% of subjects per fold), ensuring that model performance was assessed on previously unseen subjects and preventing information leakage across splits. To reduce overfitting risk, we employed a combination of early stopping, learning‐rate scheduling, and regularization (dropout and weight regularization), together with augmentation (temporal shift within ± 5 samples, small‐variance Gaussian noise, and mild amplitude scaling). Augmented windows inherited the original subject ID so that the subject‐wise split constraint was preserved.

To evaluate classification performance, we systematically compared three input modalities: standalone CS‐TENG, standalone PFOS, and the integrated CS‐TENG‐PFOS dual modality sensor (Figure 5d). Classification accuracy reached 40.38% for CS‐TENG alone and 88.46% for PFOS alone, while the fused dual‐modality approach achieved a substantial 5.77‐percentage‐point improvement to 94.23%—validating the superiority of multimodal integration for enhanced feature extraction and pattern recognition. Feature attribution analysis (Table S3) indicates that the multimodal accuracy gain is primarily driven by time‐domain gait dynamics (variability) with secondary contributions from PFOS spectral energy cues, supporting the complementarity between plantar‐pressure loading patterns and muscle activation rhythms.

Muscle activation dynamics and rhythm stability across motor states were quantified using metrics including median power frequency (MPF) and gait cycle coefficient of variation (CV) (Figures S37 and S38). Radar plots further visualized the multidimensional discriminative power of across‐channel features (Figure S39). The t‐SNE visualization (Figure 5e) reveals distinct clustering of six cohorts in the latent space, with PD's disease patients stratified into three pathophysiological subtypes: tremor‐dominant (PDTD), akinetic‐rigid (PDAR), and postural instability and gait difficulty (PDPIGD). These subtypes are all clinically established motor phenotypes and are widely used in clinical practice and research on movement disorders [64, 65, 66]. The confusion matrix (Figure 5f) confirms the model's exceptional multiclass accuracy and robustness.

Clinically, the system quantified pharmacological interventions efficiency (Figure 5g) and rehabilitation devices impacts (Figure 5h), revealing marked improvements in step frequency (from 0.8 to 0.9 Hz), step interval variability (from 19.63% to 12.51%), gait symmetry (from 0.41 to 0.85), and peak‐to‐peak amplitude (from 32.75 to 23.28, Table S4)—underscoring its utility for objective rehabilitation assessment.

To address post‐stroke depression‐induced motivational deficits, we also developed a human‐machine interaction (HMI) rehabilitation module (Figure S40). PFOS components mounted on fingers enable patients to control short‐video playback and navigate miniature vehicles (Videos S4–S6). Through repeated, task‐oriented active motor training, patients can enhance motor‐related representations of the sensorimotor system and induce experience‐dependent neural plasticity (such as enhanced cortical activation and functional reorganization), thereby supporting motor learning and promoting the recovery of motor function [67, 68, 69]. The HMI‐assisted practice enhances immersion for patients with neurological disorders during rehabilitation, avoiding the monotony of deliberate practice. This approach demonstrates potential for integration with smart assistive devices, supporting personalized, motivating, and functional recovery strategies.

## 3 Discussion

In summary, this study introduces PFOS components and a CS‐TENG component to form a WDMS that monitors human joint motions and integrates with a wireless remote healthcare platform, enabling real‐time visualization and analysis on mobile devices. Distinct from previously reported dual‐mode or TENG‐based gait systems that typically monitor single‐site signals under externally powered architectures, the WDMS realizes a self‐powered, dual‐mode, multi‐site framework that directly links plantar loading and distributed muscle strain to AI‐derived, clinically interpretable gait biomarkers and interactive rehabilitation functions. Each PFOS component features a simple three‐layer structure (POF, PU fiber, and POF), realizing strain sensing based on the Beer‐Lambert law. Since it features high stretchability and rapid responsiveness to changes in amplitude with duration, the designed sensor can detect both subtle muscle strains and large joint deformations. The experimental results show excellent performance across the full sensing range (0%–100% strain), high linearity (R^2^ = 0.96) and sensitivity (12.26 mV/%), making it well‐suited for comprehensive biomechanical monitoring. The CS‐TENG component effectively captures triboelectric signals and maintains stable output at a contact frequency of 3 Hz for more than one hour, highlighting its stable operation under continuous cycling conditions. A custom miniaturized circuit board was developed to support dual functions: energy harvesting and gait sensing, which are responsible for charging an integrated lithium battery to provide a continuous energy supply and recording the gait signals, respectively. The mode‐switching function can be conveniently toggled either by a physical button or remotely through a smartphone. Validated on both healthy subjects and patients with Parkinson's disease and stroke, the system accurately classified neurological disorders using a CNN‐LSTM model and objectively tracked rehabilitation outcomes. Beyond clinical monitoring, the WDMS also supports interactive rehabilitation tasks, enhancing patient engagement. To further improve wearability and adaptability for long‐term daily use, future iterations will pursue a fully flexible and conformal implementation by adopting elastomeric/textile substrates and compliant encapsulation for the insole‐integrated CS‐TENG, migrating rigid electronics to flexible printed circuits, and optimizing soft interconnects and strain‐relief layouts to minimize any potential motion constraints.

Overall, the WDMS lays the foundation for a next‐generation wearable healthcare platform—capable of enabling real‐time physiological monitoring, disease risk prediction, and personalized rehabilitation support. The WDMS is especially promising for patients with neurological disorders, such as Parkinson's disease and stroke, and shows broad applicability in digital healthcare. For future remote healthcare deployment, we will implement privacy‐preserving and secure remote transmission (data minimization with coded IDs, encryption/access control) and improve robustness via offline‐first buffering and automatic reconnection under intermittent connectivity. In addition, the current system has limitations that include potential long‐term signal drift and mechanical fatigue under prolonged cycling, limited cohort size, and possible performance degradation when generalizing to new users, sensor placements, or real‐world environments. In long‐term daily use, PU/elastomeric waveguides may undergo slow baseline drift due to moisture uptake/swelling, creep, and interfacial aging [70], while triboelectric outputs can attenuate under high humidity via charge dissipation and gradual surface wear/contamination [71]. These real‐life risks motivate our planned barrier encapsulation and strain‐relief packaging, together with drift‐tracking and compensation in both hardware calibration and AI preprocessing for sustained deployment. Future upgrades will incorporate drift compensation and accelerated durability testing, expand multi‐center datasets with cross‐domain validation, and optimize low‐power operation and energy management to extend battery life. Furthermore, the WDMS architecture is compatible with additional bio signal modalities. Future work will involve integrating multimodal sensors to detect temperature, humidity, and electromyography signals, enabling comprehensive multi‐site physiological data acquisition for advanced clinical applications.

## 4 Materials and Methods

### 4.1 Fabrication of the PFOS Components

Components A and B of Clear Flex 30 (Smooth‐On, USA; Silicone Elastomer Kit) were first mixed and stirred thoroughly at a volume ratio of 1:1, then placed in a defoamer (Research Air Technology, Nanjing, Jiangsu Province, China, YK‐A1) to defoam for 2 min, and then injected into the PTFE tubes. Subsequently, the PTFE tubes containing uncured PFOS fibers were fixed on an iron plate, and two plastic fibers were inserted at each end of the PFOS fibers. Manual adjustments were made to align the centerline of the plastic fibers with the centerline of the PFOS fibers; the core diameter of the plastic fibers needed to be smaller than that of the PFOS fibers. After alignment, use adhesive tape to fix the plastic fiber and PFOS fibers on the iron plate. After the PFOS fibers were cured (60°C, 5 h), the PTFE tube was peeled off, and the Part A and Part B reagents of Dragon Skin 20 (Silicone Elastomer Kit, Smooth‐On, USA) were mixed thoroughly at a 1:1 volume ratio and placed in a defoamer to defoam for 2 min. The mixture was uniformly coated on the surface of the PFOS fibers using a glass rod and left for 2 h to obtain the PU fibers. For the optical waveguiding design, the refractive index of cured Clear Flex 30 is *n*_core_ = 1.48822 (20°C) (1.48649 at 25°C, per manufacturer data), while Dragon Skin 20 silicone has a lower refractive index of approximately *n*_clad_ ≈ 1.41. This *n*_core_ > *n*_clad_ configuration forms a step‐index soft waveguide that satisfies the total internal reflection (TIR) condition.

### 4.2 Fabrication of CS‐TENG

Wear clean plastic gloves before placing two acrylic boards (160 mm × 80 mm × 2 mm) onto a clean and dry experimental platform. Spray alcohol evenly on both sides of the acrylic boards and wipe them clean with paper towels. Then, cut a copper film (200 mm × 100 mm × 50 µm) with an adhesive backing. Carefully press the copper film onto the surface of one acrylic board using a scraper to ensure smooth adherence. Fold excess copper film along the edges to the back side of the acrylic board, embedding a copper wire along one selected long edge during the folding process to serve as an electrical lead. Repeat the same process for another acrylic board. Next, cut a transparent PTFE film (200 mm × 100 mm × 50 µm). Select one copper‐coated acrylic board with the copper film side facing upward, and apply the PTFE film onto the copper surface, pressing firmly with a scraper to avoid air bubbles or wrinkles. Wrap excess PTFE film around the edges to the backside of the acrylic board. Afterward, cut a Kapton polyimide film (200 mm × 170 mm × 0.2 mm) and lay it flat on the laboratory bench. Attach one acrylic board (adhesive side down) centrally onto the Kapton film, aligning its long edge parallel to the wide side of the Kapton film. Place the second acrylic board (wrapped side down) precisely above the lower acrylic board, aligning the edges carefully. Fold the Kapton film inward toward the center and secure the top and bottom edges with adhesive tape, thus completing the assembly of the CS‐TENG board.

### 4.3 Structure and Working Principle of the Optical Signal Demodulation Circuit Board

To acquire the signal from the PFOS components in real‐time, a self‐powered circuit board for the demodulation of optical signals was designed and fabricated independently.3 light sources and receivers are on the circuit board for connecting to the PFOS components, and the poles of the circuit board are connected to the upper and lower poles of the CS‐TENG board. The light is emitted by a light‐emitting diode model SFH756 on the circuit board. One end of the optical fiber is inserted into the light‐emitting diode by plugging and unplugging to achieve laser coupling. The other end of the optical fiber is connected to the photodiode model SFH250 in the same manner so that the photodiode receives the light passing through the PFOS component. At the light source, light passes from the plastic fiber through the polymer sensing section. The receiving end at the other end reads the light signal and converts it to an electrical signal. The power generated by the CS‐TENG board can be collected by the collector circuit inside the board by repeatedly pressing the CS‐TENG board, which can supply power to the board after reaching the threshold value. At the same time, through the MOS tube to realize the Bluetooth wireless transmission, using the cell phone APP can send switching commands, and then through the Bluetooth transmission to the circuit board. The STM32 on the board controls the on/off commands of the pins so that the CS‐TENG board can be switched from power supply mode to sensing mode, so that the CS‐TENG board can be used as a pedometer to record the number of steps taken by the wearer.

To collect the optical signal of the PFOS components and the voltage signal generated by CS‐TENG in real time, a multimodal sensing signal acquisition and data‐processing circuit board was independently designed and fabricated. A rectifier‐booster circuit was implemented to capture the weak voltage output from the CS‐TENG and enable battery charging. In this design, modulated optical signals from three PFOS components were generated using three groups of infrared LEDs (SFH756), and subsequently received by three corresponding groups of photodiodes (SFH250). These photodiodes converted optical intensity signals into analog voltage signals. These analog signals, along with voltage signals generated by pressing the CS‐TENG, were input into an analog‐to‐digital converter (ADS1256). The PFOS strain channels were digitized using the 24‐bit, 8‐channel ADS1256 at a sampling rate of 10 Hz, enabling µV‐level resolution and yielding smooth raw waveforms. The printed circuit board (PCB) employed two single‐pole double‐throw CMOS analog switches (SGM3157) to switch between the sensing mode and the charging mode of the CS‐TENG. Switching signals were transmitted from a smartphone application to the microcontroller (STM32F103C8) through a Bluetooth serial interface. For wireless streaming, each frame contains 8‐channel double‐precision data (64 bytes) plus a frame identifier and checksum (67 bytes in total) transmitted at 10 Hz, corresponding to ∼6.7 kbps, which is below the 9600‐baud serial link budget; thus, throughput‐induced packet loss is not expected. Moreover, wireless transmission was used for all 60 subjects, and no link disconnections were observed during walking tests. In sensing mode, the CS‐TENG served as a sensing element, capturing gait signals. In charging mode, the AC voltage generated by the CS‐TENG was rectified and regulated by a buck‐rectifier chip (LTC3588) to provide a stable DC output for charging a lithium‐ion battery.

### 4.4 Construction and System Integration of the WDMS

After fabrication, the WDMS was assembled into a footwear‐based platform to enable synchronized capture of plantar loading and distributed muscle strain during gait. The CS‐TENG was fully encapsulated within a PU insole and mechanically coupled to foot–ground contact/separation, while the processing/demodulation board and lithium battery were mounted on the shoe upper to avoid constraining high‐deformation regions. Three PFOS components were conformally attached to targeted lower‐limb muscle groups using soft fixation to ensure stable strain transfer on curved anatomical surfaces during walking tasks. The PFOS modules were connected to the board via plastic optical fiber terminals, whereas the CS‐TENG output and power lines were electrically interfaced to the same board, thereby forming a unified hardware backbone for multimodal sensing and on‐body operation. System‐level integration was realized by combining energy management and signal acquisition within a mode‐selectable workflow on the board. Briefly, the CS‐TENG served as a shared module for both energy harvesting and plantar sensing: in the energy‐harvesting configuration, the triboelectric output was routed to the on‐board power‐management and storage path to charge the battery and sustain the system supply; in the sensing configuration, the CS‐TENG electrical signal was routed to the acquisition path as the plantar gait channel. Mode switching could be triggered either locally via a physical button or remotely via smartphone commands through the wireless link, allowing flexible selection between charging and sensing during experiments. Importantly, the PFOS demodulation and the multichannel acquisition pipeline remained consistent across modes, ensuring that the WDMS can operate as a self‐powered wearable platform with a standardized sensing interface. For multichannel acquisition, the three PFOS demodulated outputs and the CS‐TENG sensing output were digitized under a unified timing control and packaged into time‐aligned data frames for wireless streaming. In this work, the sampling rate for gait monitoring was set to 10 Hz to provide stable waveforms and robust long‐cycle operation. Each transmitted frame contained synchronized multichannel data together with an identifier/check field to ensure reliable reconstruction on the receiver side, enabling real‐time visualization and storage on a smartphone or host device. The resulting time‐aligned multimodal sequences were used directly as the standardized input to the CNN–LSTM network, facilitating multimodal fusion of plantar loading and multi‐site strain dynamics without additional post hoc alignment across sensors. For wearable safety, the CS‐TENG remained electrically isolated from the wearer through complete encapsulation within the PU insole, ensuring that no electrode was in direct contact with skin during prolonged use.

### 4.5 Gait‐Based Neurological Disorder Recognition via CNN‐LSTM Neural Networks

In this study, “clinical validation” is based on the doctor's diagnosis as the reference standard, comparing the outputs of the neural network with clinical diagnostic labels to verify whether the system exhibits the ability to distinguish between groups consistent with clinical classification. Specifically, we collected gait data from 60 subjects, including individuals from neurological disease cohorts (such as PD and stroke‐related gait abnormalities) and control groups. The true labels for each subject were sourced from clinical diagnostic records of a cooperating hospital, and these labels were determined prior to model training and evaluation to ensure they were independent of the algorithm's output. To achieve this validation, we first applied overlapping sampling to the collected gait signal data, with a window length of 300. The resulting windows were evaluated using subject‐wise grouped cross‐validation. In each fold, approximately 80% of subjects were used for training, and the remaining 20% of subjects were held out for testing, with all windows from the same subject kept within a single split to avoid information leakage. The training set was used to optimize the parameters of the CNN‐LSTM network model. This model consists of two parallel branches: the plantar pressure signal branch extracts feature through two convolutional layers, each followed by batch normalization and pooling operations; the surface electromyography (sEMG) signal branch employs a three‐layer convolutional structure for feature extraction. The outputs of the two branches are concatenated along the channel dimension and subsequently passed through an LSTM network to capture temporal dynamics. Finally, these features are fed into a fully connected layer followed by a Softmax output layer for classification. During training, the Adam optimizer is adopted, and a learning rate decay strategy is incorporated to dynamically adjust the learning rate. By comparing the outputs of the neural network model with clinical diagnostic results, we are able to validate the system's accuracy and reliability for practical clinical applications.

### 4.6 Characterization and Measurement

The mechanical properties of the PFOS components were tested using an SRI multidimensional force transducer (Sunrise Instruments, Nanning 530007, China; 45499 Irvine Dr, Novi, MI 48374, USA, M3705C). A stepping motor (Zhejiang Wenzhou Pufide, TB6600) was used to generate the tensile strain of the PFOS components. The performance of the CS‐TENG was characterized using linear motors (6514, Dongfang Zhongke, China; PS01‐37*120‐C, LinMot, USA).

### 4.7 Ethics Statement and Statistical Analysis

This study involved human gait data collection. All procedures involving human participants were conducted in accordance with the Declaration of Helsinki and were approved by the Ethics Committee of Yangxin County Traditional Chinese Medicine Hospital (Approval No. 20250701). Written informed consent was obtained from all participants prior to testing. Data processing and statistical analyses were performed using MATLAB R2023b, Origin 2022, and GraphPad Prism 9.5. Unless otherwise stated, device characterization experiments were conducted once under the specified conditions; calibration tests were repeated three times, and sensitivity and goodness‐of‐fit (R^2^) were obtained by linear regression. For the rehabilitation assessment (Pre vs. Post) gait features, two‐tailed paired‐samples t‐tests were applied, and the mean difference (Post–Pre), 95% confidence interval, and *p*‐value were reported. Statistical significance was defined as *p* < 0.05.

## Author Contributions

T.L.L. and C.Z. conceived this project. Q.A.W. performed conceptualization, data curation, formal analysis, methodology, visualization, wrote the original draft, and reviewed and edited the final manuscript; H.L. and G.Y.L. performed conceptualization, data curation, visualization and data curation; G.X.L. and G.Y.L. performed visualization, wrote the original and review and edited the final manuscript; G.X.L., Q.A.W., H.L., G.Y.L., Y.X., J.W., Z.Q.W., F.Z., and F.L.L. performed data curation and formal analysis; H.L., Q.A.W., and F.L.L. performed methodology; G.X.L. performed formal analysis; Q.A.W., G.X.L., H.T.Z., G.Y.L. and F.L.L. performed visualization. T.L.L. and C.Z. acquired resources and funding, supervision, wrote the original review, and edited the manuscript. Yan Xu, Jun Wang, Chuanbing Yu, Jun Liu, and Xujiang Chen assisted in acquiring patient data from the hospital.

## Conflicts of Interest

The authors declare no conflicts of interest.

## Supporting information




**Supporting File 1**: advs74685‐sup‐0001‐SuppMat.docx.

**Supporting File 2**: advs74685‐sup‐0002‐VideoS1.mp4.

**Supporting File 3**: advs74685‐sup‐0003‐VideoS2.mp4.

**Supporting File 4**: advs74685‐sup‐0004‐VideoS3.mp4.

**Supporting File 5**: advs74685‐sup‐0005‐VideoS4.mp4.

**Supporting File 6**: advs74685‐sup‐0006‐VideoS5.mp4.

**Supporting File 7**: advs74685‐sup‐0007‐VideoS6.mp4.

## Data Availability

The data that support the findings of this study are available from the corresponding author upon reasonable request.
