# Supplementary material for: AI‐Assisted Self‐Powered Wearable Dual‐Mode Sensor With TENG and Stretchable Optical Fiber for Neurological Disorder Diagnostics
Source: Adv Sci (Weinh). 2026 Mar 12;13(27):e22179. doi: 10.1002/advs.202522179 (PMC13170242; doi:10.1002/advs.202522179)
Supplement: Supplementary file 1 — Supporting File 1: advs74685‐sup‐0001‐SuppMat.docx. [file ADVS-13-e22179-s002.docx]

Supporting Information for

**AI-Assisted Self-Powered Wearable Dual-Mode Sensor with TENG and Stretchable Optical Fiber for Neurological Disorder Diagnostics**

Tianliang Li, *et al.*

*Corresponding author. Email: E-mail addresses: qianaowang@whut.edu.cn, zlwang@binn.cas.cn, czhang@binn.cas.cn

**This PDF file includes:**

Figs. S1 to S40

Movies S1 to S6

**Other Supporting Information for this manuscript include the following:**

Movies S1 to S6


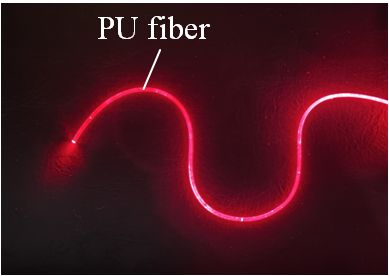


Figure S1

**The PU fiber illuminated by a red laser.**


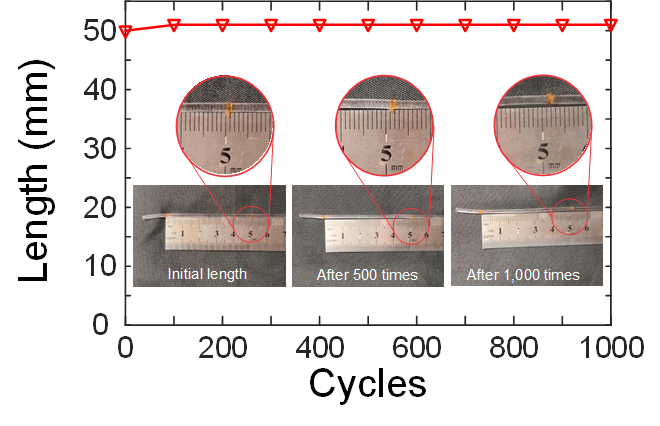


Figure S2

**Mechanical durability of PU fibers during cyclic stretching.** Fiber lengths were measured at the initial state, after 500 cycles, and after 1,000 cycles of stretching.


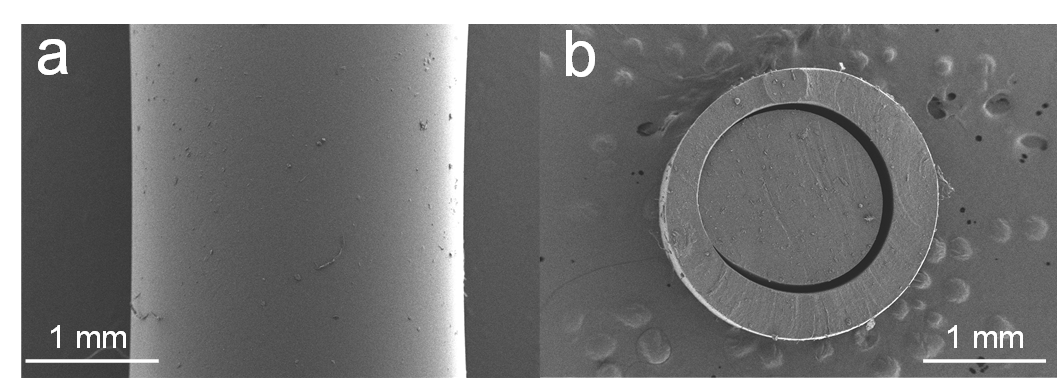


Figure S3

**Optical micrographs of the PU fiber with cladding.** (a) Front view. (b) Top view.


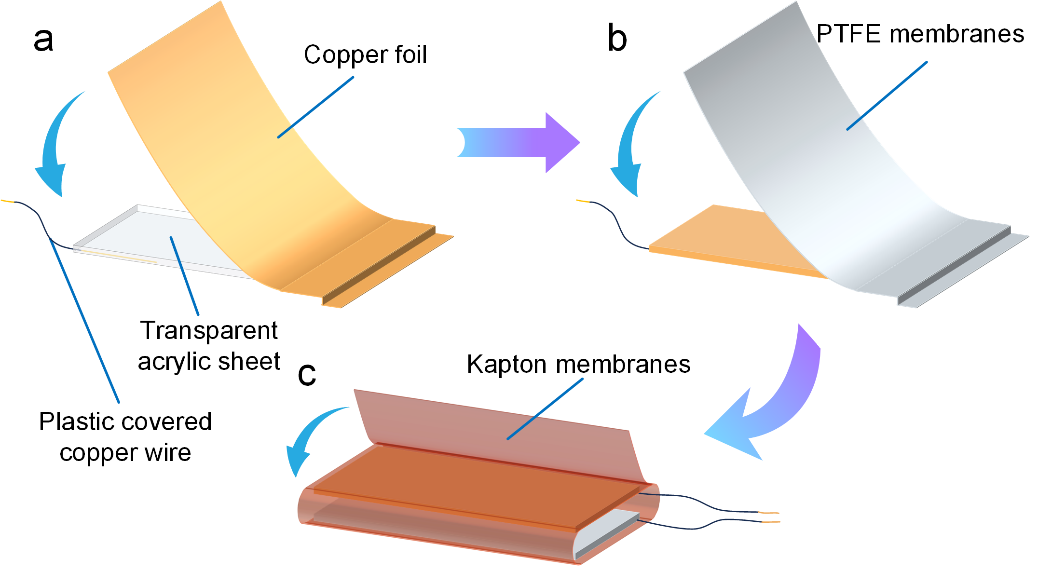


Figure S4

**Schematic illustration of the fabrication process of CS-TENG.** (a) Covering copper foil on the upper surface of an acrylic sheet, embedding an insulated copper wire, and repeating to prepare two copper-clad acrylic sheets (b) Wrapping a PTFE film around one copper-clad acrylic sheet. (c) Encapsulating the assemblies with Kapton film.

**
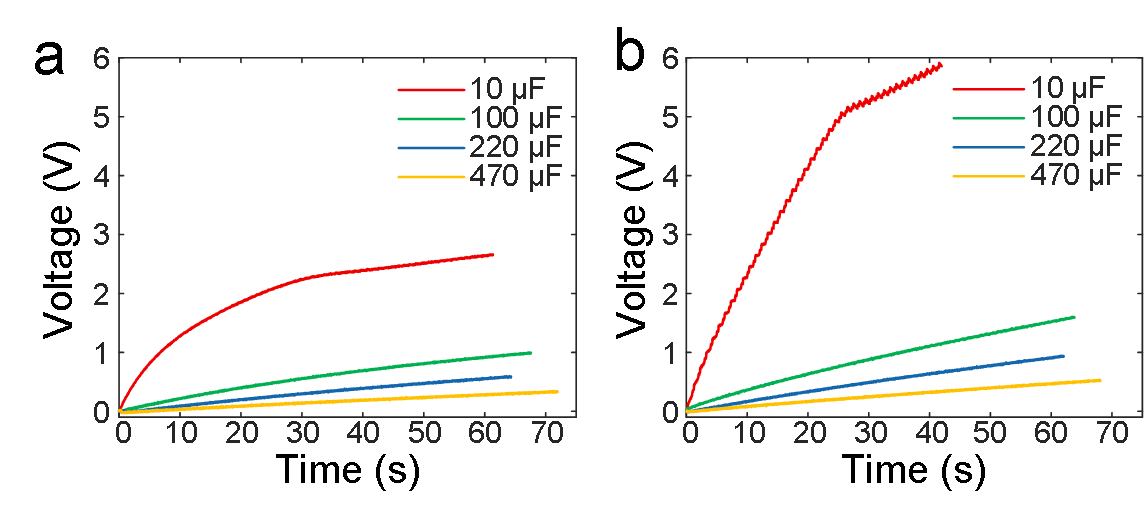
**

Figure S5.

**Charging performance of different capacitors without and with a power management circuit**: (a) without a management circuit; (b) with a management circuit.

**
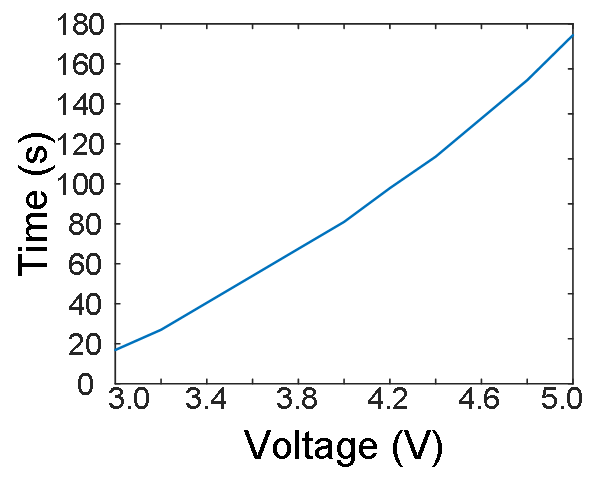
**

Figure S6.

**The discharge time of capacitors storing charged electricity at different voltages.**


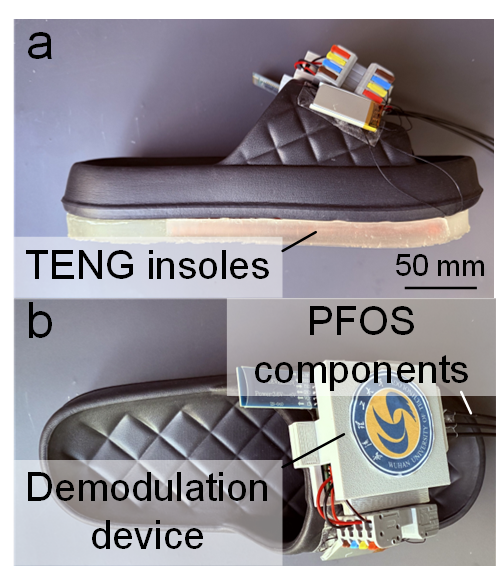


Figure S7.

**Assembly of the smart gait-acquisition shoe.** (a) Smart sole structure with an internally encapsulated CS-TENG component and external wiring. (b) Upper structure of smart shoe integrating three PFOS components and a demodulation circuit.


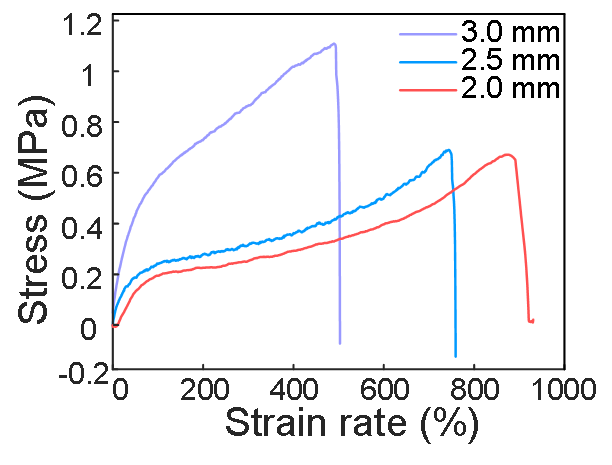


Figure S8.

**Stress−strain curves of PU fibers with different diameters.**


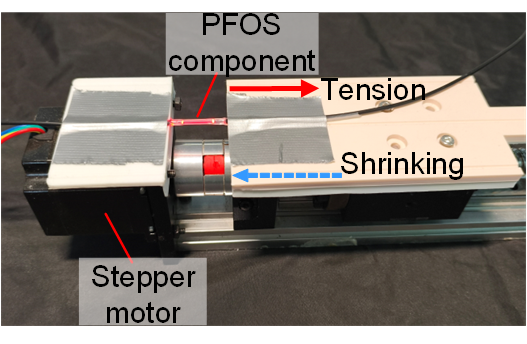


Figure S9.

**Calibration setup for the PFOS component, using a stepper motor for controlled actuation.**


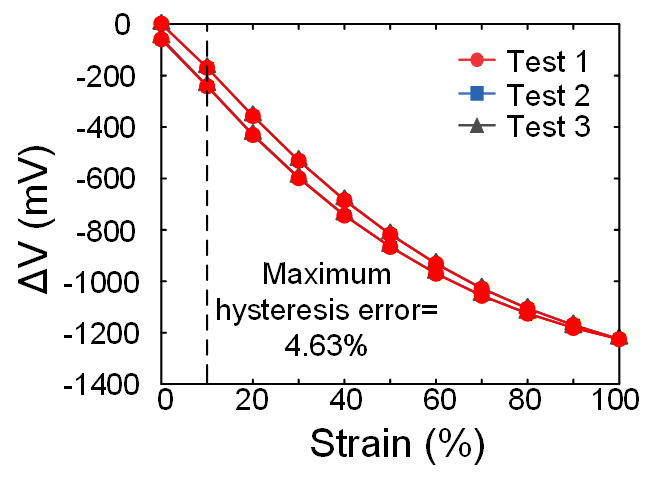


Figure S10.

**Three static calibrations of the PFOS component over 0–100% strain.** The dashed guides highlight the maximum separation between the loading and unloading outputs at the same strain point; the resulting maximum hysteresis error is 4.63%, normalized to the full-scale output.

**
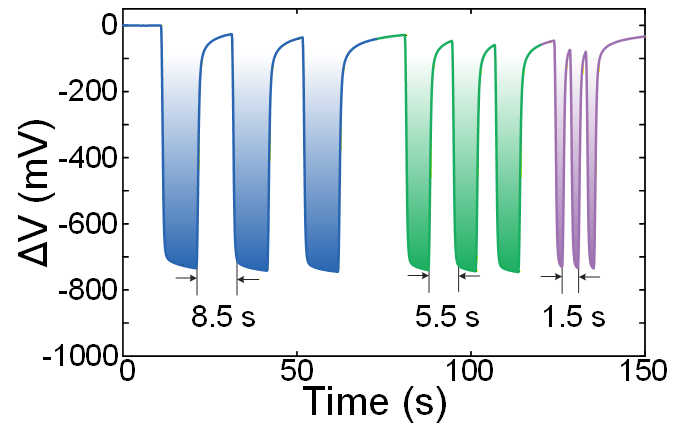
**

Figure S11.

**Signal stability of the PFOS component during three equal-strain stretching cycles with intervals of 8.5 s, 5.5 s, and 1.5 s.**

**
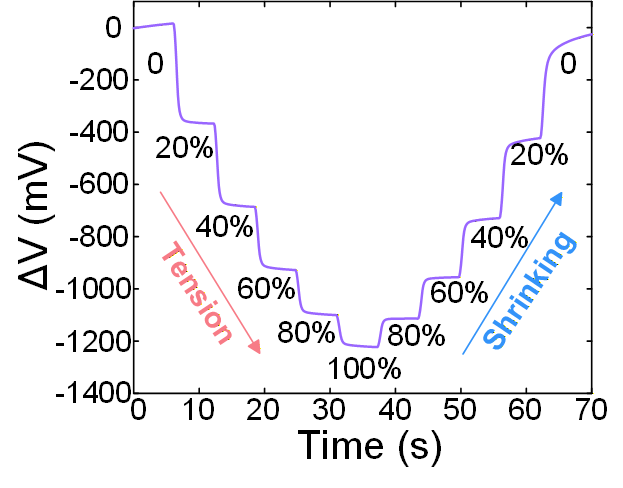
**

Figure S12.

**Signal changes of the PFOS component at different stretch rates.** From 0 to 100%, interval stretching 20% and hold for a while, then return to 0 in the same way.


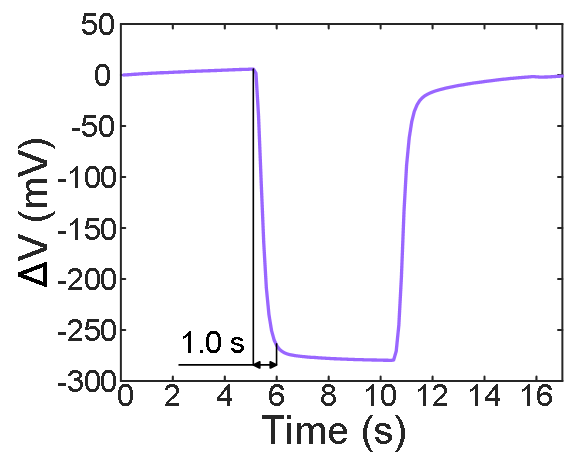


Figure S13.

**Experimentally measured response time of** **the PFOS component.**


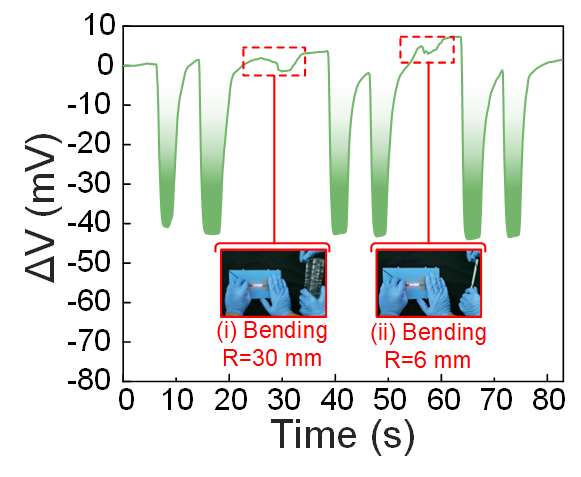


Figure S14.

**Characterization of the PFOS component signal variation under controlled bending conditions.** (ⅰ) 30 mm bend radius. (ⅱ) 6 mm bend radius.


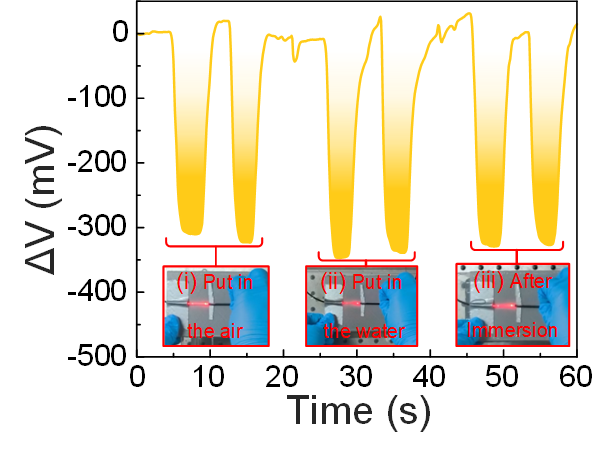


Figure S15.

**Water resistance characterization of the PFOS component: (**ⅰ**) Baseline signal in air, (**ⅱ**) Real-time response during water immersion, (**ⅲ**) the PFOS component performance after water soaking.**


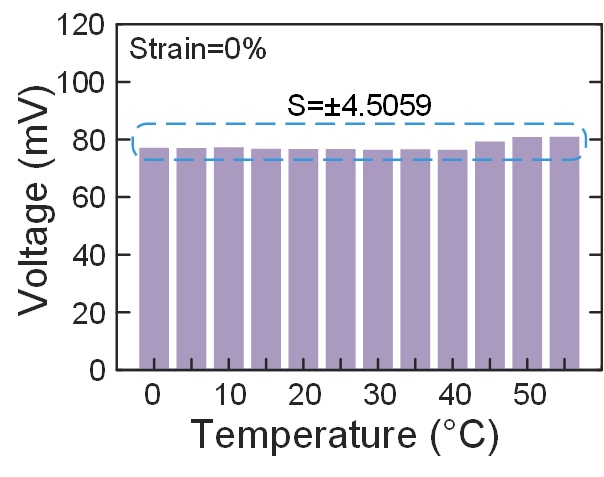


Figure S16.

**Temperature-dependent signal variation of the PFOS component in aqueous environments at zero-strain condition** **(S represents standard deviation).**

**
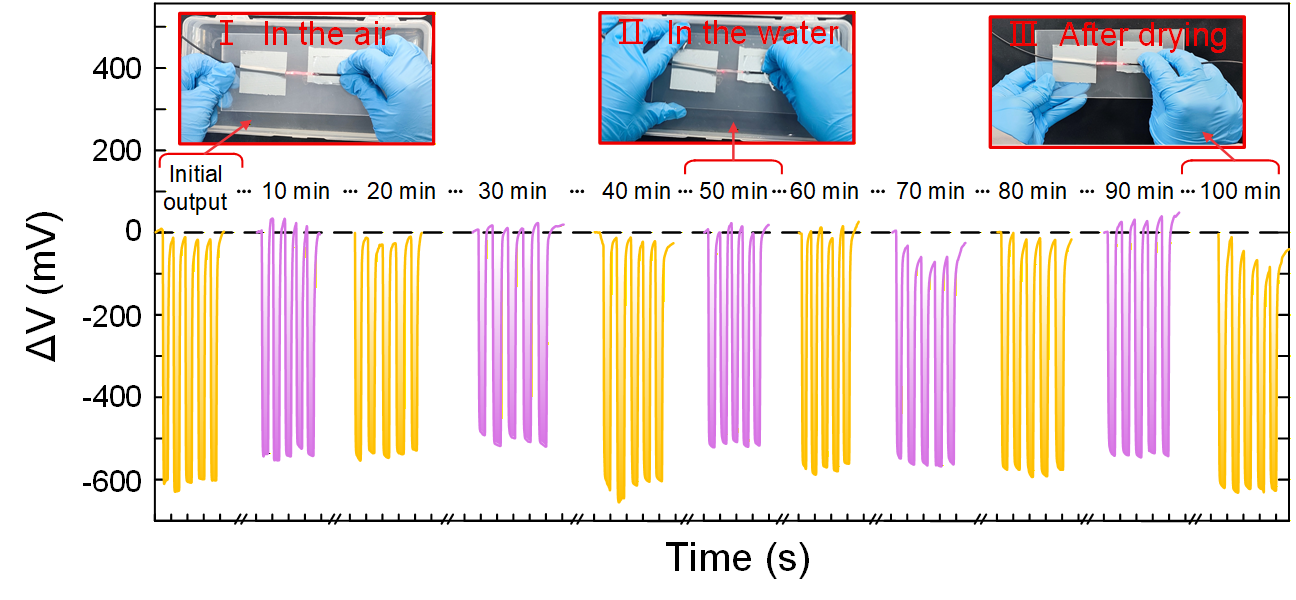
**

Figure S17.

**Dry–wet cycling stability of the PFOS component.** The PFOS component was subjected to alternating dry–wet cycling under identical tensile loading. It was first stretched for five identical stretch–release cycles in air (I, In the air), then immersed in water and stretched for another five cycles under the same tensile condition (II, In the water). After 10 min of immersion, the component was removed, naturally air-dried, and stretched for five additional cycles (III, After drying). The immersion–drying procedure was repeated for five rounds in total (0–100 min). The ΔV waveforms and amplitudes remain stable across all rounds, indicating that water exposure has no appreciable effect on the PFOS output and demonstrating robust waterproof operation.

**
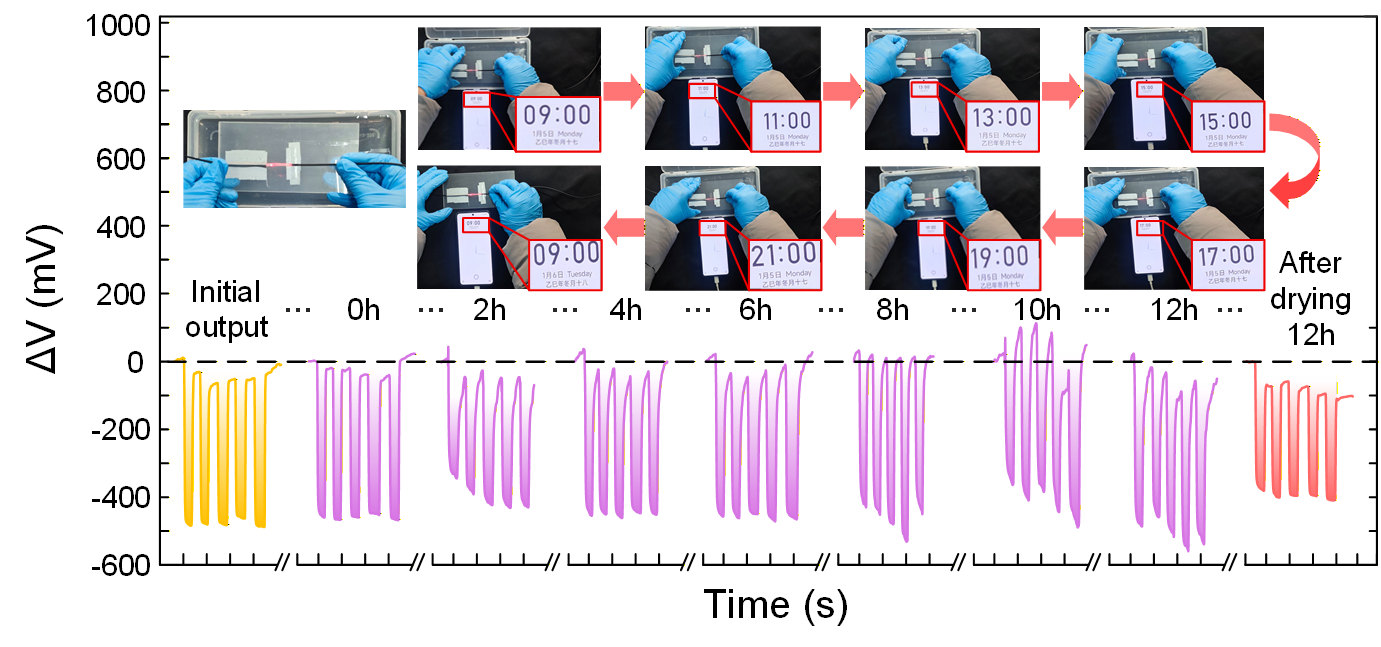
**

Figure S18.

**Output stability of the PFOS component under simulated sweat exposure.** To mimic sweat contact, a 0.4 wt% saline solution (with ionic strength close to human sweat) was used as the test medium. The component was first stretched for five identical stretch–release cycles in dry conditions to obtain the initial output (Initial output), then immersed in saline for 2 h, removed, and stretched for another five cycles under the same tensile loading. This immersion–test–re-immersion procedure was repeated until the cumulative exposure time reached 12 h (0–12 h, with an additional “After drying 12 h” test shown on the right). The ΔV waveforms and amplitudes remain consistent at all time points, with no noticeable baseline drift or attenuation, demonstrating strong tolerance to sweat-like conditions and robust long-term stability.


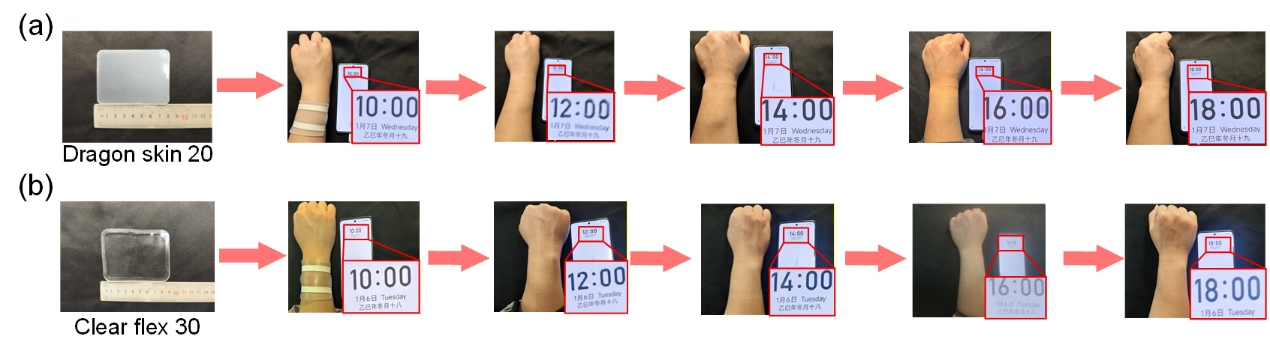


Figure S19.

**Preliminary skin-compatibility assessment under prolonged contact of elastomeric interface films.** As a preliminary wearability/compatibility evaluation, two elastomeric films were fixed on the dorsal hand skin of a subject under otherwise identical test conditions. (a) Dragon Skin 20 film was applied to the left hand (80 mm × 60 mm × 1 mm), and (b) Clear Flex 30 film was applied to the right hand (90 mm × 70 mm × 1 mm). The skin condition was documented by photographs after 0/2/4/6/8 h of continuous contact to inspect potential erythema or irritation. No obvious erythema or irritation was observed on either side, suggesting good short-term skin compatibility of these elastomer interfaces on this time scale.

**
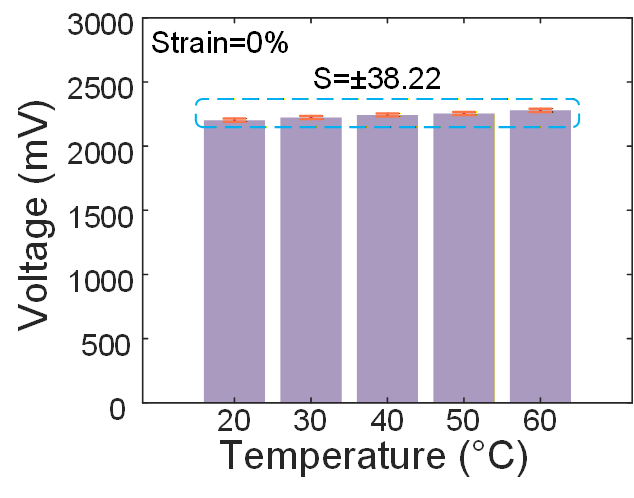
**

Figure S20.

**Temperature-cycling stability of the PFOS component.** The PFOS component was placed in a temperature chamber and cycled between 20 and 60 °C for three rounds while continuously monitoring the raw output voltage. At zero strain (Strain = 0%), the temperature-induced variation is reversible and repeatable (highlighted by the dashed box), indicating no irreversible drift under thermal perturbation. Moreover, the strain response remains stable before and after cycling under identical tensile loading, demonstrating robust reliability and repeatability during temperature cycling (S represents standard deviation).

**
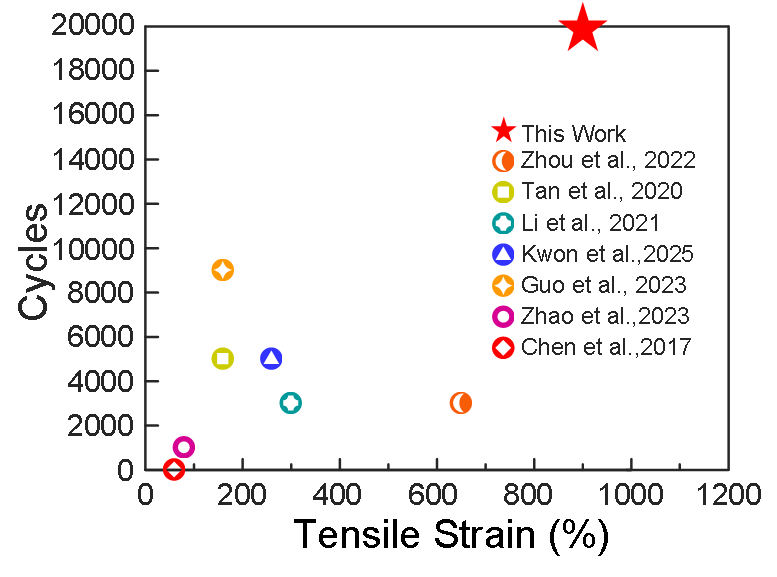
**

Figure S21.

**Tensile strain performance comparison between this work and state-of-the-art sensors across key metrics.**

**Table S1. Quantitative comparison of maximum tensile strain (stretchability) and fatigue durability of representative stretchable devices**

| Ref. | Max tensile strain / stretchability | Fatigue durability |
| --- | --- | --- |
| This work | **900%** | **20,000** |
| [57] | 650% | 3,000 |
| [58] | 160% | 5,000 |
| [59] | 300% | 3,000 |
| [60] | 260% | 5,000 |
| [61] | 160% | 9,000 |
| [62] | 80% | 1,000 |
| [63] | 60% | / |

[57] J. Zhou, X. Long, J. Huang, C. Jiang, F. Zhuo, C. Guo, H. Li, Y. Fu, H. Duan, *npj Flexible Electron.* **2022**, *6*, 55.

[58] C. Tan, Z. Dong, Y. Li, H. Zhao, X. Huang, Z. Zhou, J.-W. Jiang, Y.-Z. Long, P. Jiang, T.-Y. Zhang, B. Sun, *Nat. Commun.* **2020**, *11*, 3530.

[59] H. Li, Y. Zhang, Y. Wu, H. Zhao, W. Wang, X. He, H. Zheng, *Beilstein J. Nanotechnol.* **2021**, *12*, 402.

[60] J. H. Kwon, H. C. Moon, *Adv. Funct. Mater.* **2025**, 2503935.

[61] X. Guo, W. Hong, Y. Zhao, T. Zhu, L. Liu, H. Li, Z. Wang, D. Wang, Z. Mai, T. Zhang, J. Yang, F. Zhang, Y. Xia, Q. Hong, Y. Xu, F. Yan, M. Wang, G. Xing, **2023**.

[62] R. Zhao, Y. He, Y. He, Z. Li, M. Chen, N. Zhou, G. Tao, C. Hou, *ACS Appl. Mater. Interfaces* **2023**, *15*, 16063.

[63] X. Chen, K. Parida, J. Wang, J. Xiong, M.-F. Lin, J. Shao, P. S. Lee, *ACS Appl. Mater. Interfaces* **2017**, *9*, 42200.


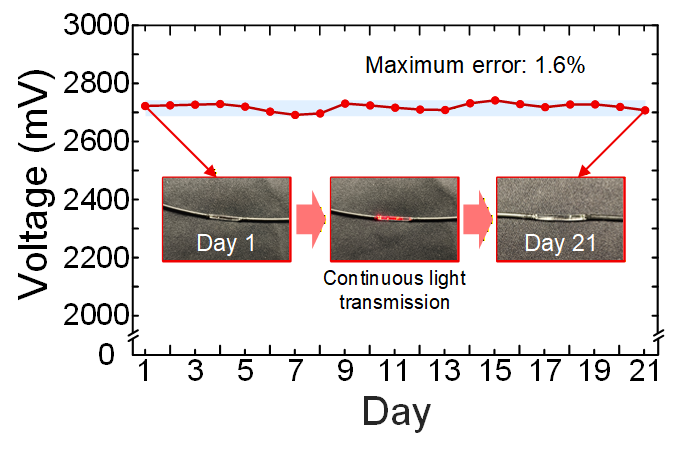


Figure S22.

**Baseline optical stability of the PFOS component under continuous illumination aging (21 days).** To assess potential photo-oxidative degradation/yellowing of the polyurethane (Clear Flex 30) PFOS core under prolonged light exposure, the PFOS component was aged under continuous LED illumination for 21 days (3 weeks). The baseline output voltage at 0% strain was recorded once per day at a fixed time (17:00, Beijing time). The baseline voltage was 2758 mV on day 1 and 2745 mV on day 21. The maximum baseline fluctuation over the entire period was 44 mV, corresponding to an error of 1.60%, with no noticeable baseline drift or abnormal attenuation. These results indicate minimal illumination-induced changes on the time scale tested, supporting the long-term optical stability and Beer–Lambert-based quantitative reliability of the PFOS component.


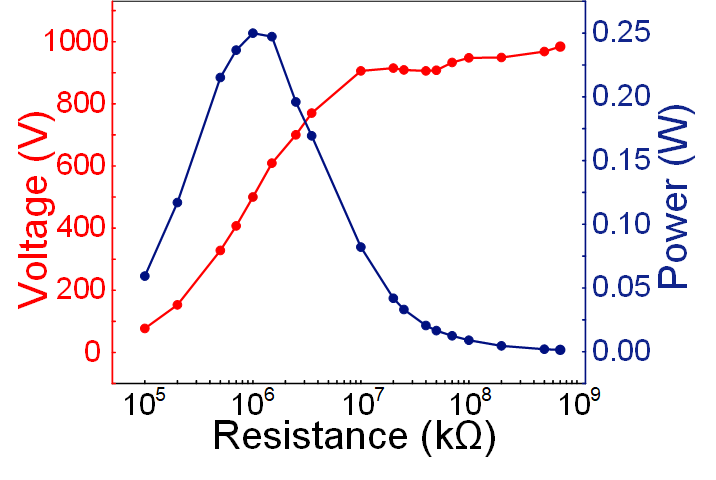


Figure S23.

**Output voltage characteristics of CS-TENG under impedance matching optimization.**


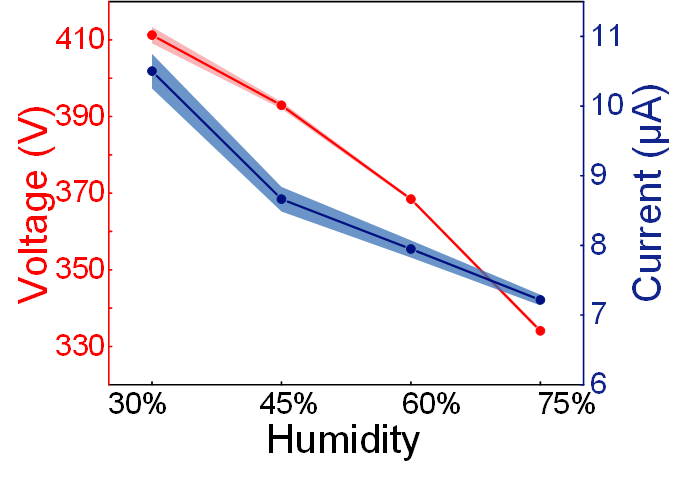


Figure S24.

**Humidity sensing characteristics: Simultaneous current and voltage response of circuit board across 30-75% RH.**


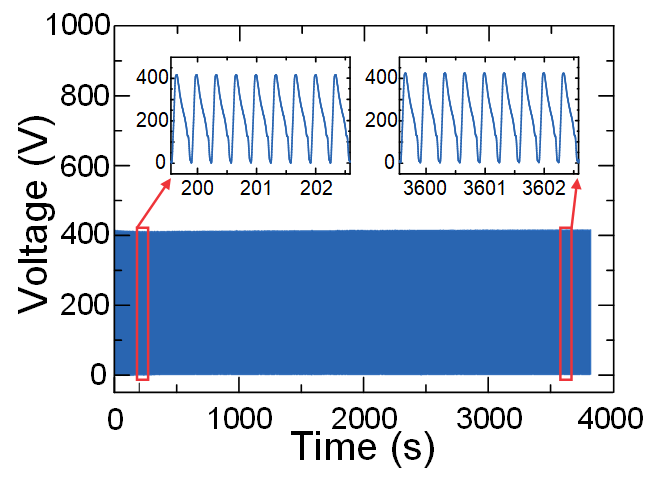


Figure S25.

**Voltage output durability under sustained operation: TENG performance during 60 minutes of continuous mechanical cycling.**


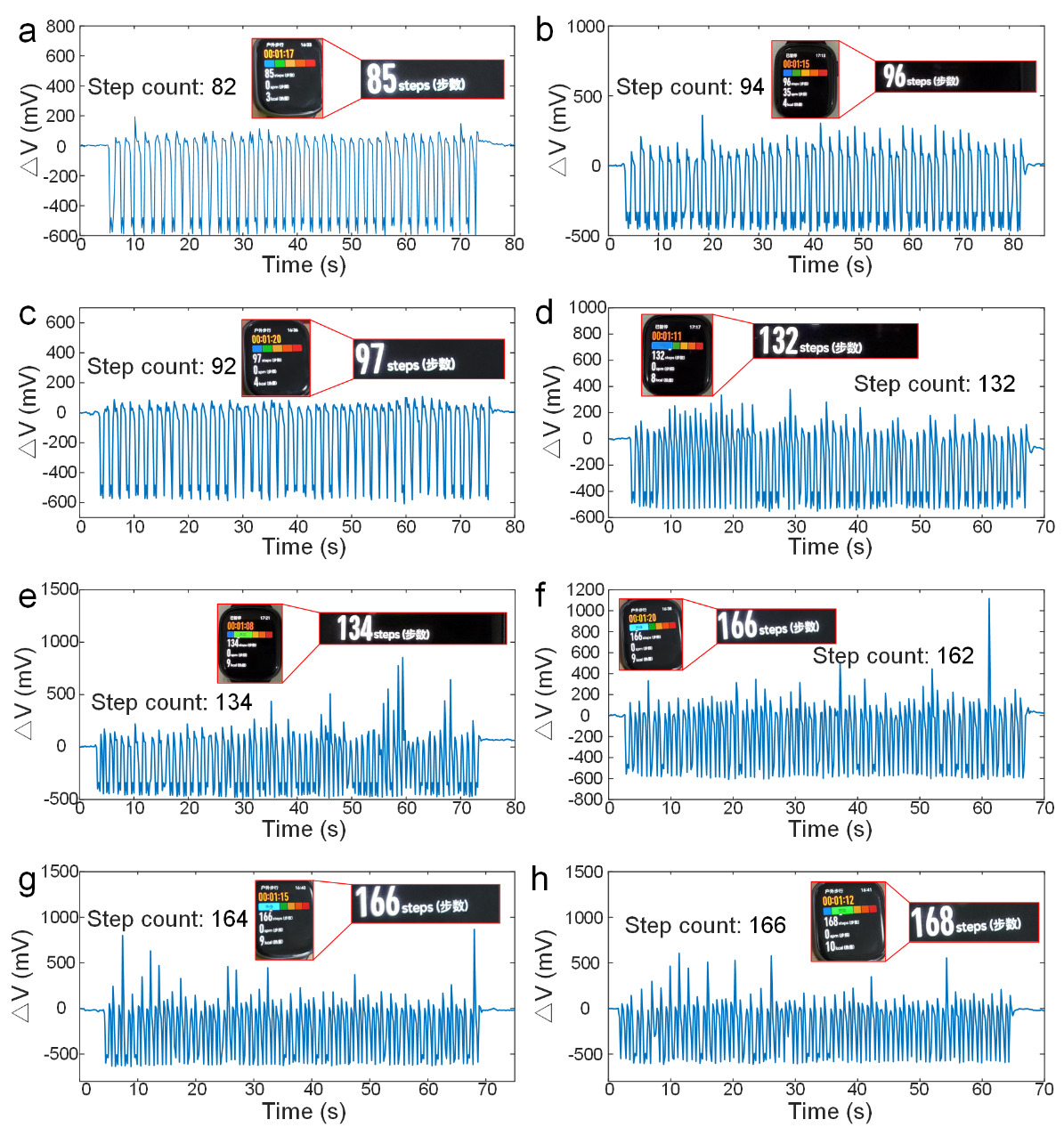


Figure S26.

**Source data for the remaining 8 gait patterns and schematic of the wearable sensor bracelet data acquisition.**


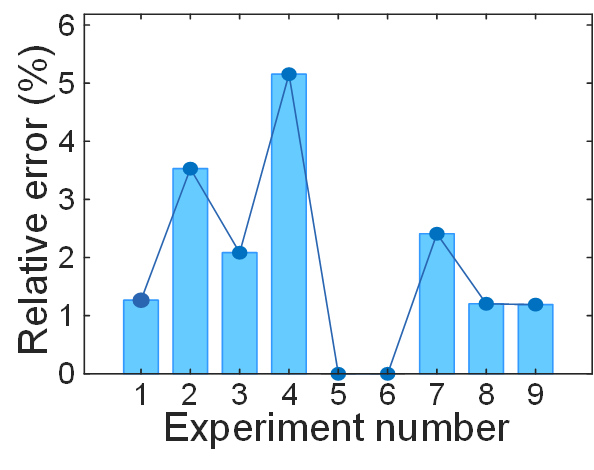


Figure S27.

**Distribution of measurement errors across 9 comparative trials.**


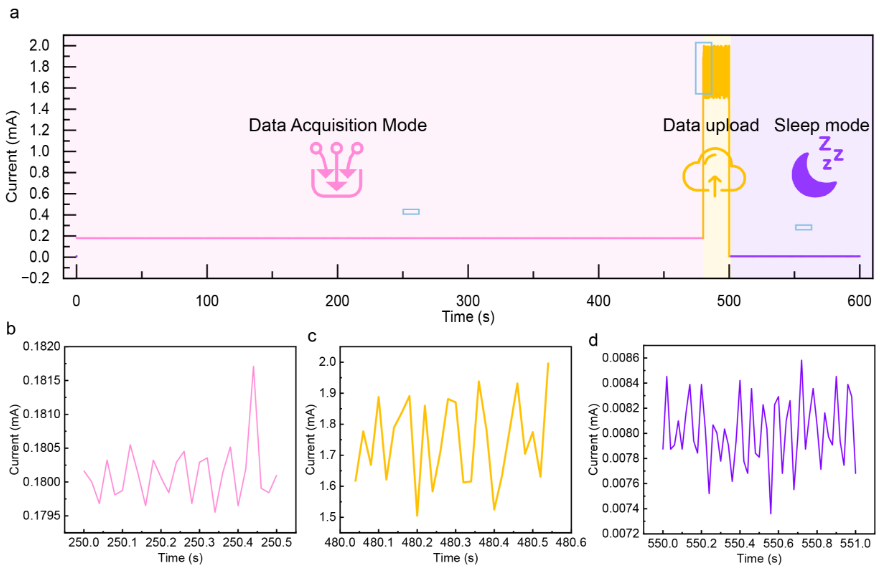


Figure S28.

**System operating current in data acquisition mode, data upload mode and sleep mode.**

**Table S2. Power consumption for the single operating cycle (600 s)**

| **Working mode** | **Duration (s)** | **Current (mA)** | **Power (mW)** | **Energy consumption (mJ)** |
| --- | --- | --- | --- | --- |
| Data acquisition | 480 | 0.2 | 0.64 | 307.2 |
| Data upload | 20 | 1.8 | 5.76 | 115.2 |
| Sleep mode | 100 | 0.007 | 0.0224 | 2.24 |
| Total | 600 | -- | -- | 424.64 |


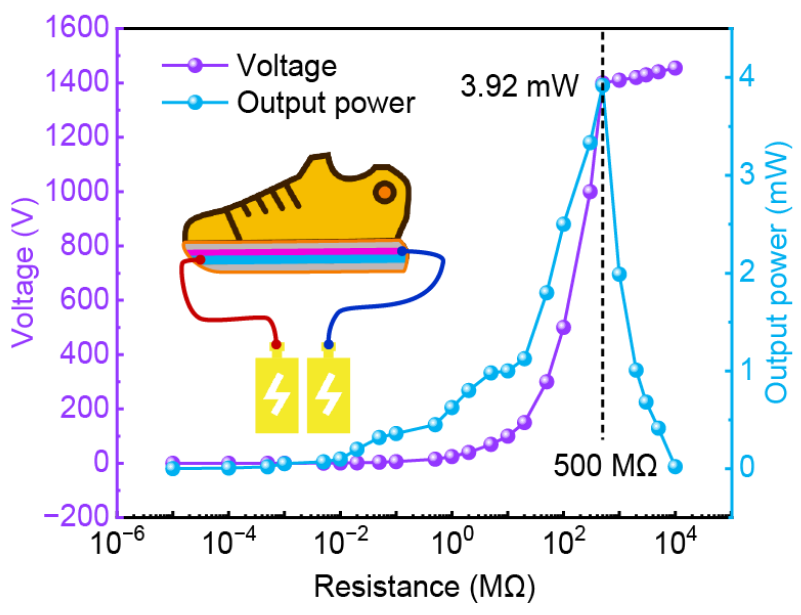


Figure S29.

**CS-TENG's power matching test shows an output power of 3.92 mW at 500 MΩ external resistance.**


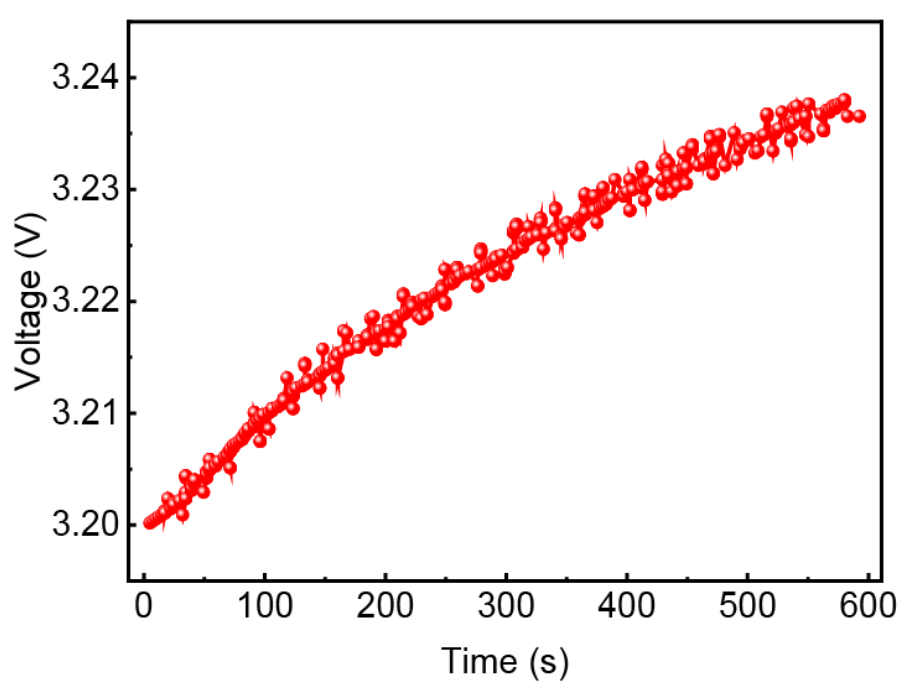


Figure S30.

**The voltage variation across the battery after 10 minutes of system operation.**

**
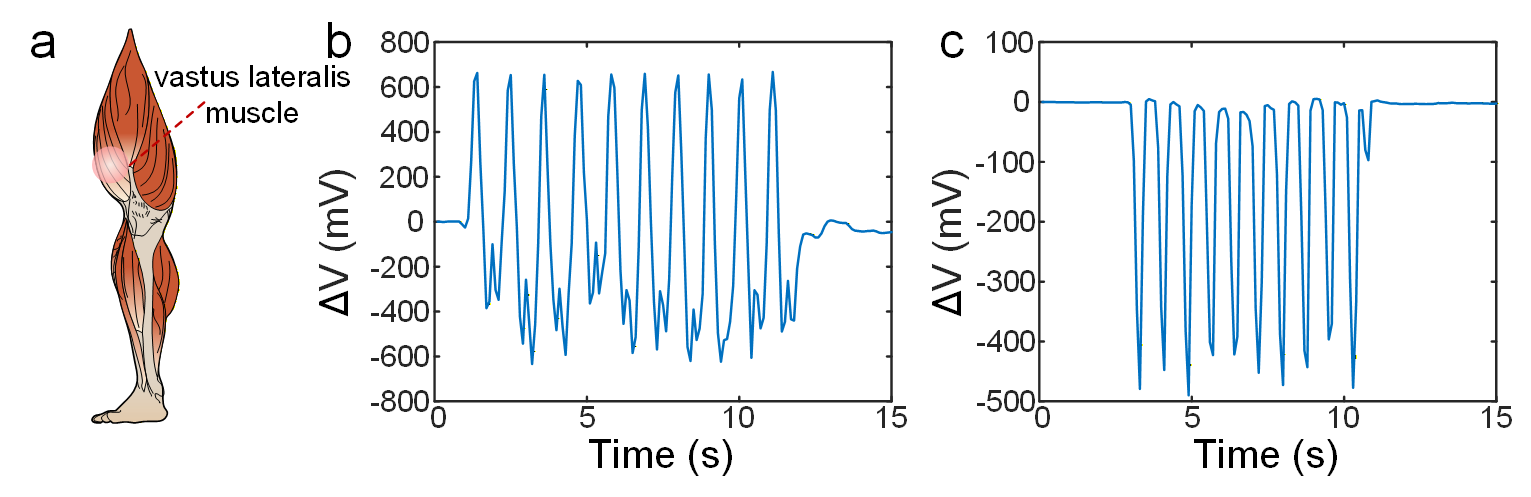
**

Figure S31.

**Detection of vastus lateralis muscle movements using the PFOS component:** (a) Schematic of the vastus lateralis muscle, (b) Sensor signal response during toe standing, (c) Sensor signal response during walking.


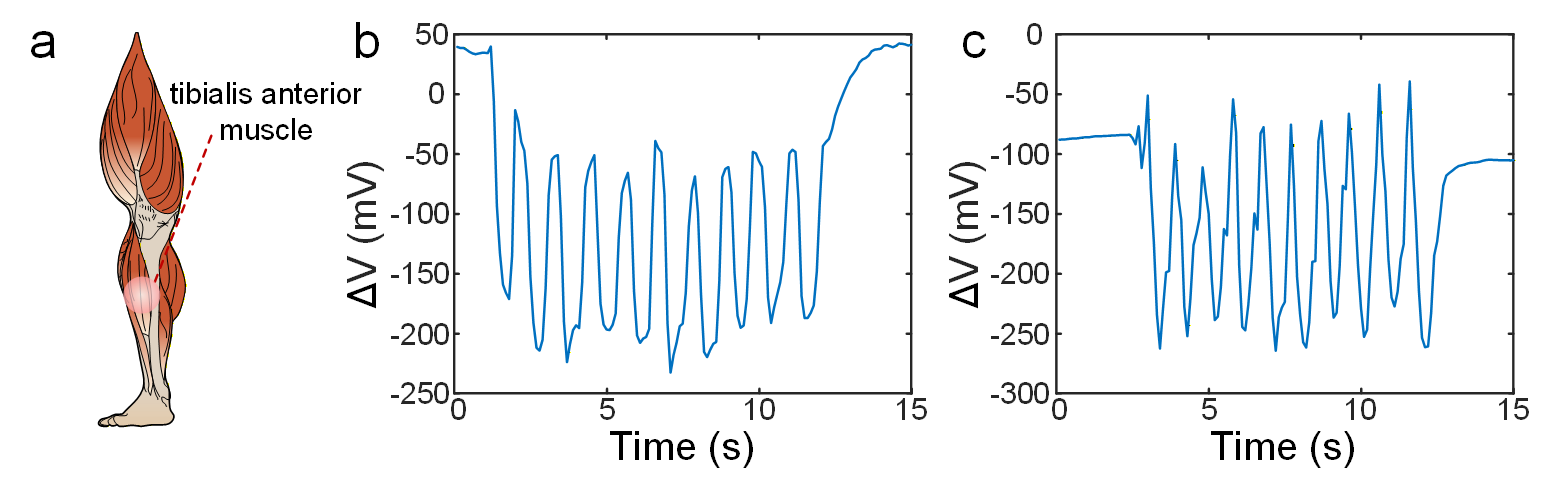


Figure S32.

**Detection of tibialis anterior muscle movements using the PFOS component:** (a) Schematic of the tibialis anterior muscle, (b) Sensor signal response during toe standing, (c) Sensor signal response during walking.


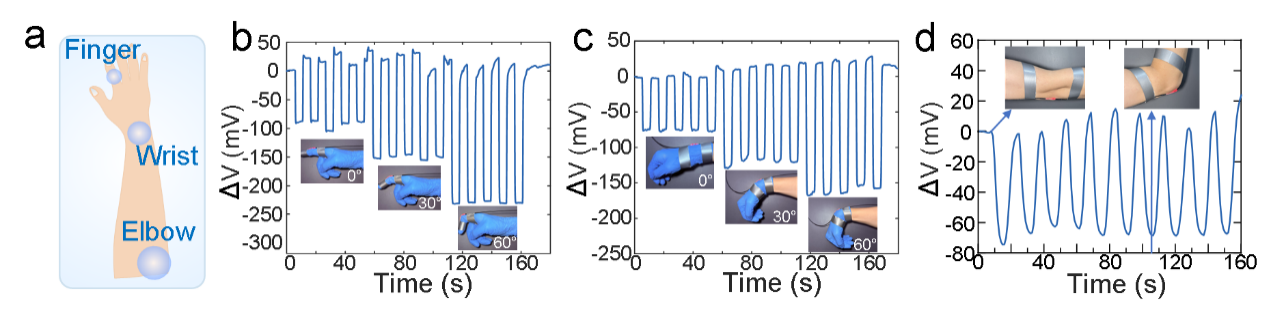


Figure S33.

**Sensor placement and strain testing for subtle muscle movement detection using the PFOS component:** (a) Schematic of sensor placement on the hand, (b) Strain testing with sensor on finger, (c) Strain testing with sensor on wrist, (d) Strain testing with sensor on elbow.


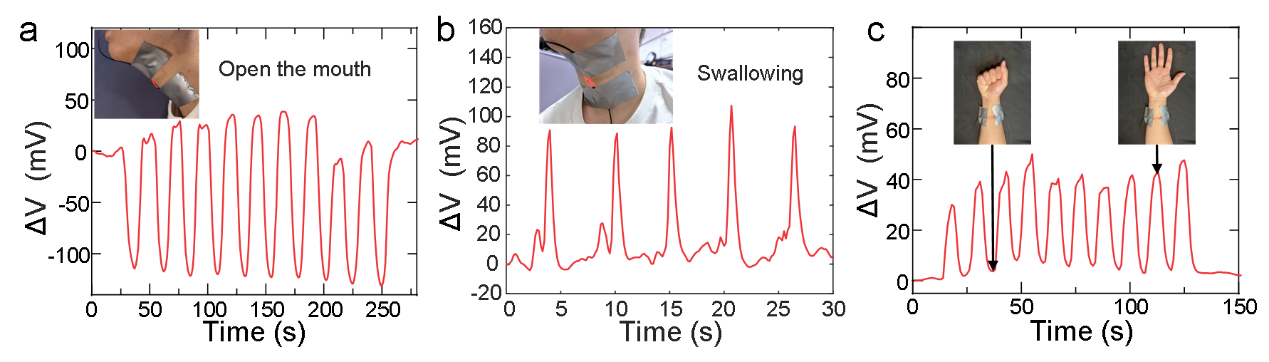


Figure S34.

**Detection of subtle muscle movements using the PFOS component:** (a) Signal response to mouth opening, (b) Signal response to swallowing, (c) Signal response to fist clenching.


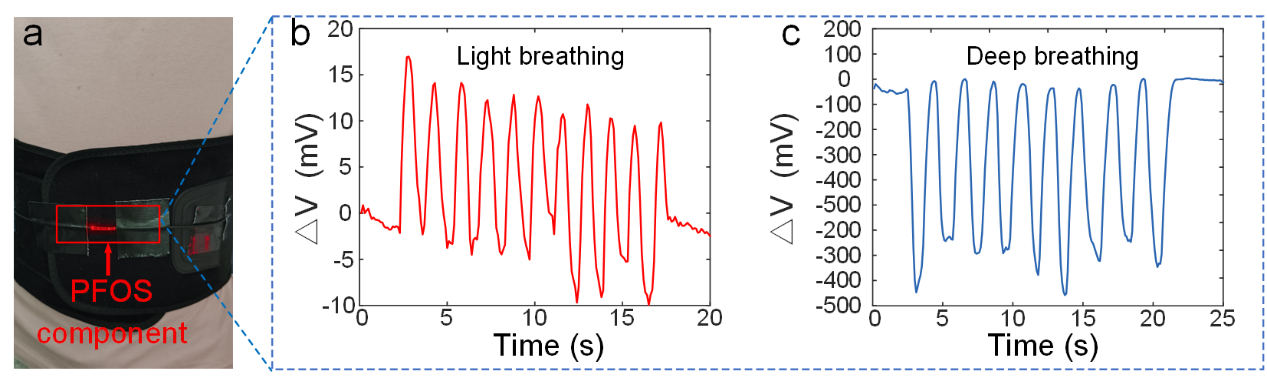


Figure S35.

**Detection of breathing movements using the PFOS component:** (a) Photograph of the PFOS component fixed on the abdomen. (b) Signal changes during light breathing. (c) Signal changes during deep breathing.


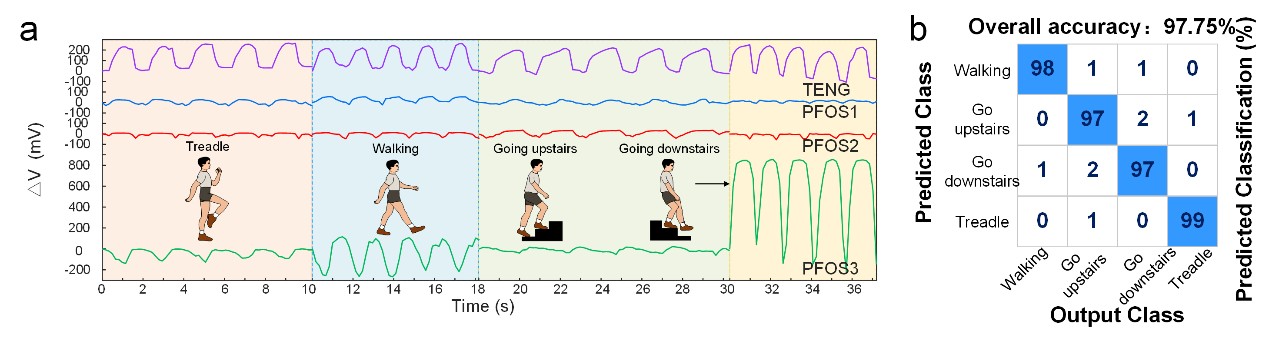


Figure S36.

**Dual-modal sensing experiments in normal human subjects:** (a) Signal change curves for different gait states. (b) Confusion matrix for gait state recognition accuracy.

**Table S3. Engineered feature inventory and attribution analysis for multimodal gait classification.**

We extracted 49 engineered features per channel (20 time-domain, 17 frequency-domain, 5 time–frequency, and 7 wavelet features), yielding 196 features for the dual-modality input (4 channels: ch0 = CS-TENG plantar pressure; ch1–ch3 = PFOS lower-limb muscle/strain signals). Feature attribution was performed using RF pre-screening followed by perturbation-based permutation importance on the CNN–LSTM pipeline. For domain statistics, each feature was assigned to a single domain using the domain token immediately following the channel prefix (e.g., “ch2_tf_” counted as time–frequency only).

**(A) Feature inventory (per channel 49features)**

| Domain | No. | Feature names |
| --- | --- | --- |
| Time domain | 20 | mean, std, var, median, min, max, range, iqr, peak, peak to peak, skewness, kurtosis, rms, zero crossing rate, autocorr max, autocorr mean, diff1 mean, diff1 std, diff2 mean, diff2 std. |
| Frequency domain | 17 | spectral mean, spectral std, spectral max, dominant frequency, dominant power, spectral centroid, spectral bandwidth, low frequency energy, mid frequency energy, high frequency energy, low frequency ratio, mid frequency ratio, high frequency ratio, frequency spectral entropy, psd mean, psd max, psd peak frequency. |
| Time-frequency | 5 | tf_stft_mean, tf_stft_std, tf_stft_max, tf_time_freq_energy, tf_freq_variance, |
| Wavelet | 7 | mean of level-1 approximation coefficients, standard deviation of level-1 approximation coefficients, mean of level-1 detail coefficients, standard deviation of level-1 detail coefficients, energy of level-1 detail coefficients, mean of level-2 approximation coefficients, energy of level-2 detail coefficients |

**(B) Domain-level attribution (Top 49; dual modality; unique-domain counting)**

| Domain | No. of unique feature types | Feature names |
| --- | --- | --- |
| Time domain | 17 | diff1 mean |
| Frequency domain | 8 | low frequency energy |
| Time-frequency | 1 | tf_stft_mean |
| Wavelet | 5 | energy of level-1 detail coefficients |

**(C) Top-ranked features (dual modality)**

| Rank | Feature | Domain | Interpretability cue |
| --- | --- | --- | --- |
| 1 | ch3_time_diff1_std (PFOS) | Time | PFOS temporal irregularity / rapid fluctuation |
| 2 | ch0_time_std (CS-TENG) | Time | plantar-pressure loading variability |
| 3 | ch1_freq_mid_freq_eneygy (PFOS) | Frequency | mid-band rhythmic energy cue |
| 4 | ch1_time_skewness (PFOS) | Time | waveform asymmetry / activation pattern shift |
| 5 | ch1_time_max (PFOS) | Time | peak activation intensity |

**Table S4. Statistical Summary of Pre- and Post-Treatment Gait Features.**

| **Parameter** | **Pre-treatment (Mean±SD)** | **Post-treatment (Mean±SD)** | **Δ(Post-Pre)** | **95% Cl of Δ** | **p-value** |
| --- | --- | --- | --- | --- | --- |
| Step frequency (Hz) | 0.660 ± 0.106 | 0.727 ± 0.012 | 0.0669 | [-0.9949, 1.1287] | 0.570 |
| Step interval variability (CV, %) | 16.370 ± 1.320 | 6.607 ± 0.564 | -9.7631 | [-16.5544, -2,9719] | 0.035 |
| Symmetry index (a.u.) | 0.987 ± 0.011 | 0.967 ± 0.022 | -0.0202 | [-0.1257, 0.0853] | 0.248 |
| Peak-to-Peak amplitude (P2P, a.u./mV) | 127.335 ± 10.274 | 124.039 ± 17.255 | -3.2960 | [-250.6288, 244.0368] | 0.893 |


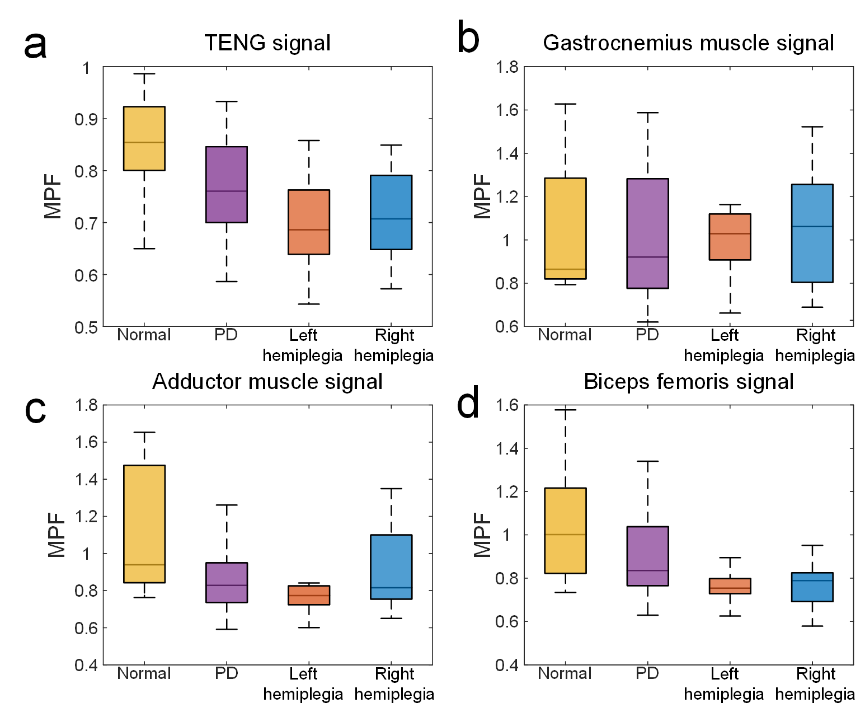


Figure S37.

**Box plots of Median Power Frequency (MPF) values for different subjects and channels:** (a) TENG, (b) Gastrocnemius, (c) Adductor, (d) Biceps femoris.


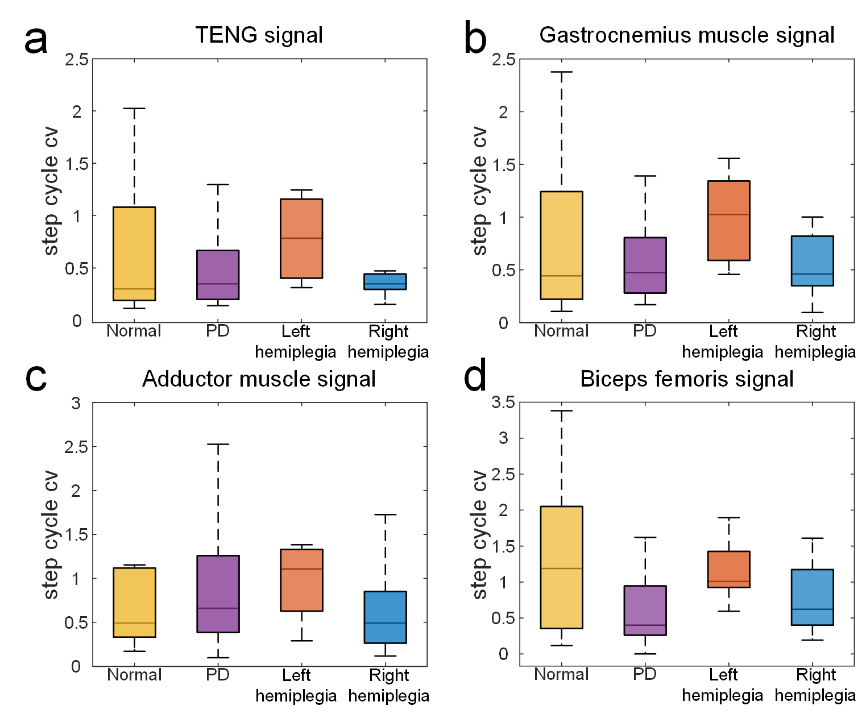


Figure S38.

**Box plots of the coefficient of variation for gait parameters derived from signals of different subjects across channels:** (a) TENG signal, (b) Gastrocnemius muscle signal, (c) Adductor muscle signal, (d) Biceps femoris signal.

**
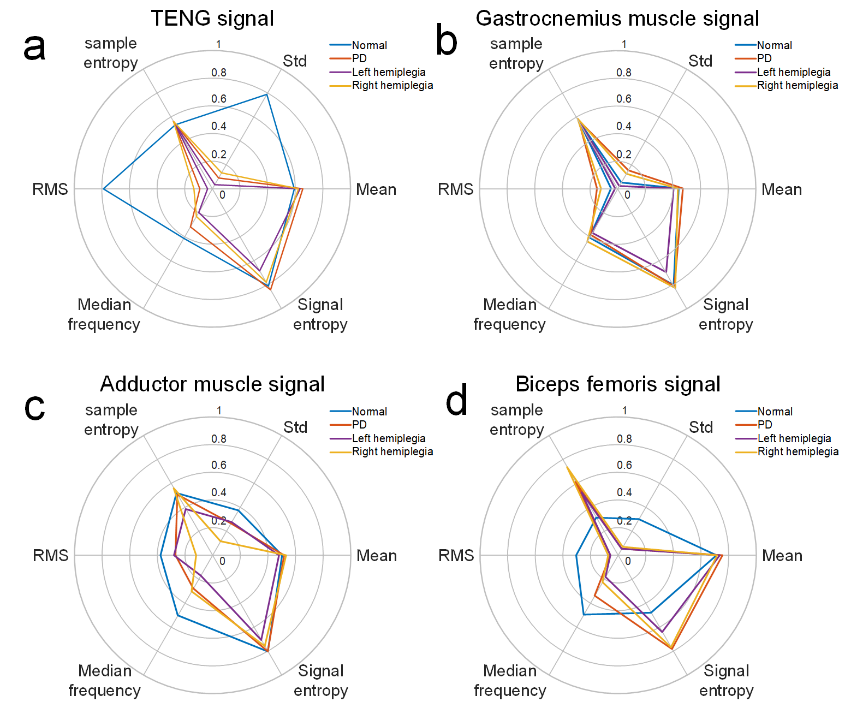
**

Figure S39.

**Radar plots depicting the signal characteristic values across different channels:** (a) TENG signal, (b) Gastrocnemius muscle signal, (c) Adductor muscle signal, (d) Biceps femoris signal.


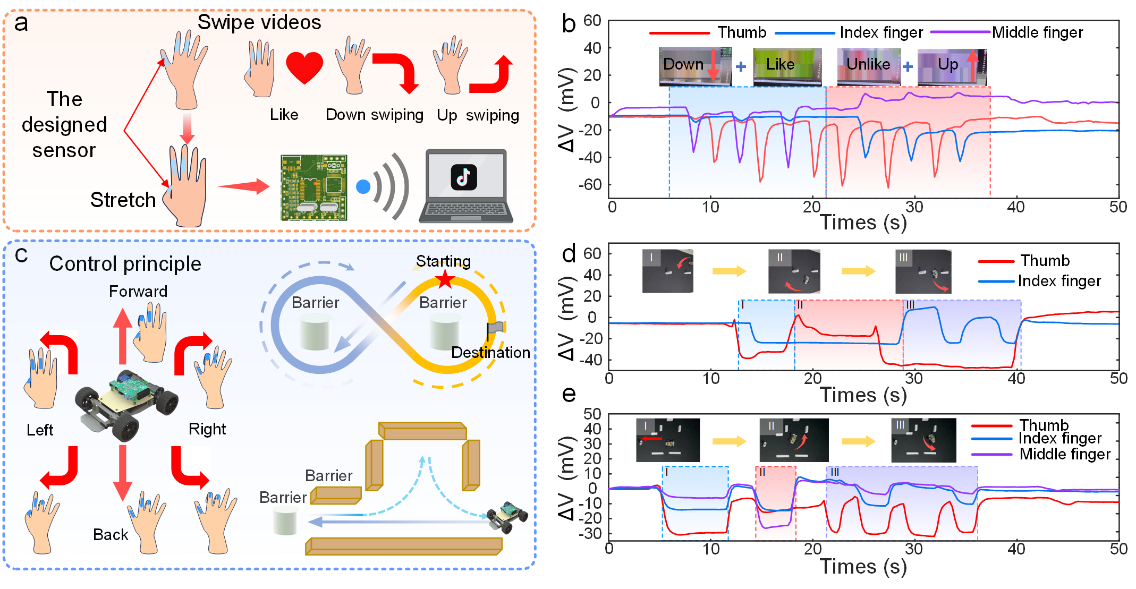


Figure S40.

**Experiment on TikTok video and car control based on the PFOS component.** (a) Schematic of computer-controlled remote operation setup. (b) Signal acquisition data from the computer control interface. (c) Gesture commands for the direction control of the car, as well as a schematic of the car's 8-shaped trajectory for obstacle avoidance and reversing into a parking space. (d) Signal data corresponding to the "8" -shaped trajectory of the trolley. (e) Signal data corresponding to the car reversing into a parking space.

Movie S1

Experiment on charging WDMS by stepping.

Movie S2

Mobile app real-time wireless monitoring of leg muscle signals.

Movie S3

Gait signal acquisition experiment based on integrated shoes.

Movie S4

Wireless TikTok video control experiment based on the PFOS component.

Movie S5

Remote control car moving in "8" shape experiment based on the PFOS components.

Movie S6

Remote control car reversing experiment based on the PFOS components.
